# Cardio- and reno-protective effects of dipeptidyl peptidase III in diabetic mice

**DOI:** 10.1016/j.jbc.2021.100761

**Published:** 2021-05-08

**Authors:** Masahiro Komeno, Xiaoling Pang, Akio Shimizu, Md Rasel Molla, Mako Yasuda-Yamahara, Shinji Kume, Nor Idayu A. Rahman, Joanne Ern Chi Soh, Le Kim Chi Nguyen, Mohammad Khusni B. Ahmat Amin, Nao Kokami, Akira Sato, Yoshihiro Asano, Hiroshi Maegawa, Hisakazu Ogita

**Affiliations:** 1 Division of Molecular Medical Biochemistry, Department of Biochemistry and Molecular Biology, Shiga University of Medical Science, Otsu, Japan; 2 Department of Emergency, The Fourth Affiliated Hospital of China Medical University, Shenyang, China; 3 Department of Medicine, Shiga University of Medical Science, Otsu, Japan; 4 Department of Cardiovascular Medicine, Osaka University Graduate School of Medicine, Suita, Japan

**Keywords:** complement, diabetic nephropathy, heart failure, peptidase, peptides, permeability, rho, AngII, angiotensin II, BP, blood pressure, DM, diabetes mellitus, DPPIII, dipeptidyl peptidase III, FITC, fluorescein isothiocyanate, GLP-1, glucagon-like peptide-1, HUVEC, human umbilical vein endothelial cell, LV, left ventricular, PBS, phosphate-buffered saline, PKC, protein kinase C, SGLT2, sodium-glucose cotransporter 2, SRM, selected reaction monitoring, T1DM, type 1 DM, T2DM, type 2 DM, WGA, wheat germ agglutinin

## Abstract

Diabetes mellitus (DM) causes injury to tissues and organs, including to the heart and kidney, resulting in increased morbidity and mortality. Thus, novel potential therapeutics are continuously required to minimize DM-related organ damage. We have previously shown that dipeptidyl peptidase III (DPPIII) has beneficial roles in a hypertensive mouse model, but it is unknown whether DPPIII has any effects on DM. In this study, we found that intravenous administration of recombinant DPPIII in diabetic db/db mice for 8 weeks suppressed the DM-induced cardiac diastolic dysfunctions and renal injury without alteration of the blood glucose level. This treatment inhibited inflammatory cell infiltration and fibrosis in the heart and blocked the increase in albuminuria by attenuating the disruption of the glomerular microvasculature and inhibiting the effacement of podocyte foot processes in the kidney. The beneficial role of DPPIII was, at least in part, mediated by the cleavage of a cytotoxic peptide, named Peptide 2, which was increased in db/db mice compared with normal mice. This peptide consisted of nine amino acids, was a digested fragment of complement component 3 (C3), and had an anaphylatoxin-like effect determined by the Miles assay and chemoattractant analysis. The effect was dependent on its interaction with the C3a receptor and protein kinase C-mediated RhoA activation downstream of the receptor in endothelial cells. In conclusion, DPPIII plays a protective role in the heart and kidney in a DM animal model through cleavage of a peptide that is a part of C3.

Diabetes mellitus (DM) is globally one of the most common chronic diseases, characterized by high blood glucose and insufficient insulin production and/or supply (1, 2). The number of DM patients is increasing in both developed and developing countries; 23 million adults have been diagnosed with DM in the United States, and there are 415 million adults with DM diagnoses globally (3, 4). DM causes severe damage in several organs and tissues, including the kidney, heart, blood vessels, and nerves (5), which frequently leads to life-threatening events, such as myocardial infarction. When DM is classified by type 1 DM (T1DM) and type 2 DM (T2DM) of which pathogenetic processes are different, mortality and incidence of cardiovascular diseases including renal dysfunction are higher in T2DM than in T1DM (6). These outcomes are also observed in younger DM patients: among T1DM and T2DM patients with similar age of onset between 15 and 30 years, T2DM is associated with a greater mortality and more cardiovascular diseases compared with T1DM (7). Thus, more intensive care and therapy are needed in T2DM patients.

Among DM-induced complications, diabetic cardiomyopathy and diabetic kidney disease (DKD) often occur from the early stage and/or short duration after onset of DM and seem to be hallmarks to presume the progression and severity of DM (8, 9). Diabetic cardiomyopathy is defined as DM-related ventricular dysfunction that occurs in the absence of coronary atherosclerosis or hypertension (10), and diastolic dysfunction is considered to be the indicator for diabetic cardiomyopathy (11). The prevalence of diastolic dysfunction is higher in patients with T2DM than in those with T1DM, suggesting an increased risk of cardiac failure in T2DM (12, 13). Albuminuria is a useful symptom to define DKD and predict its progression (14, 15). Pathologically, glomerular basement membrane thickening and mesangial matrix expansion occur in the middle to late stages of DKD, leading to renal fibrosis and glomerulosclerosis (16, 17). Similar to diabetic cardiomyopathy, the incidence of DKD in T2DM is higher than that in T1DM (18, 19).

Strict control of blood glucose is important in preventing DM-induced complications in both T1DM and T2DM (20, 21, 22, 23, 24, 25). However, based on more severe prognosis in T2DM than in T1DM even when they follow the currently available intensive blood glucose management for DM, continuing development of therapies in addition to the blood-glucose-lowering therapy is required to completely cure DM and prevent DM-related physical dysfunctions. To date, there are several reports showing that peptides derived from endogenous proteins in the serum exhibit cytotoxicity and exacerbate the prognosis of diseases (26, 27). In this context, we consider that one of the potential approaches to develop the glycemic control-independent new therapy for T2DM would be the identification of harmful circulating peptides increased in T2DM and the exploration of methods and techniques to exclude them from the circulatory system.

Dipeptidyl peptidase III (DPPIII) is an aminopeptidase that cleaves specific dipeptides from the N terminus of relatively small peptides that consist of approximately less than 12 amino acids (28). In contrast to DPPIV, which digests glucagon-like peptide-1 (GLP-1) and is a target for DM therapy, DPPIII does not cleave GLP-1 and displays no effect on glycemic control. Instead, DPPIII digests some bioactive peptides, including angiotensins, and enkephalins (29), which consist of less than eight amino acids. We have determined the kinetic characteristics of DPPIII for angiotensin II (AngII) and IV and demonstrated the blood-pressure-lowering effect of DPPIII on hypertensive mice by reducing the plasma AngII concentration (30). However, it remains unknown whether DPPIII exhibits any effects on DM.

In this study, we used the diabetic db/db mouse as a T2DM model (31) and investigated how intravenously administered DPPIII functions in the diabetic mice. Although DPPIII administration did not affect the blood glucose level, it significantly improved DM-induced cardiac and renal dysfunctions. We also identified the peptide that was cleaved by DPPIII. Intravenous injection of this peptide in db/db mice exacerbated the organ damage. Furthermore, we explored the mechanism by which this takes place.

## Results

### No change in obesity, hyperglycemia, hemodynamics, or plasma lipid profiles in db/db mice after DPPIII administration

We first examined whether DPPIII administration affected any phenotypes of the diabetic model mice. The db/db mice were intravenously administered with DPPIII (10 μg/g body weight) or phosphate-buffered saline (PBS) as a control for 8 weeks as previously described (30). As a reference, PBS-infused C57BL/6 mice were observed simultaneously. At the beginning of the experiment (0 weeks), db/db mice aged 8 weeks displayed significant obesity and hyperglycemia compared with C57BL/6 mice (Fig. S1, *A* and *B*). Body weight and the blood glucose level gradually increased in db/db mice during the experimental period, and this increase was unaffected by DPPIII treatment (Fig. S1, *A* and *B*). Hemodynamics monitored by heart rate and systolic blood pressure (BP) was also unchanged after the DPPIII injection as well as during the experimental period (Fig. S1, *C*–*F*). This is consistent with our previous findings that DPPIII-mediated BP-lowering effects were observed specifically in AngII-stimulated hypertensive mice, but not in normotensive mice (30). We further confirmed that DPPIII treatment displayed no effects on plasma cholesterol or triglyceride levels in db/db mice, which were significantly higher than those in C57BL/6 mice (Fig. S1, *G* and *H*).

### Prevention of diastolic dysfunction in the heart by DPPIII administration

**Figure 1:**
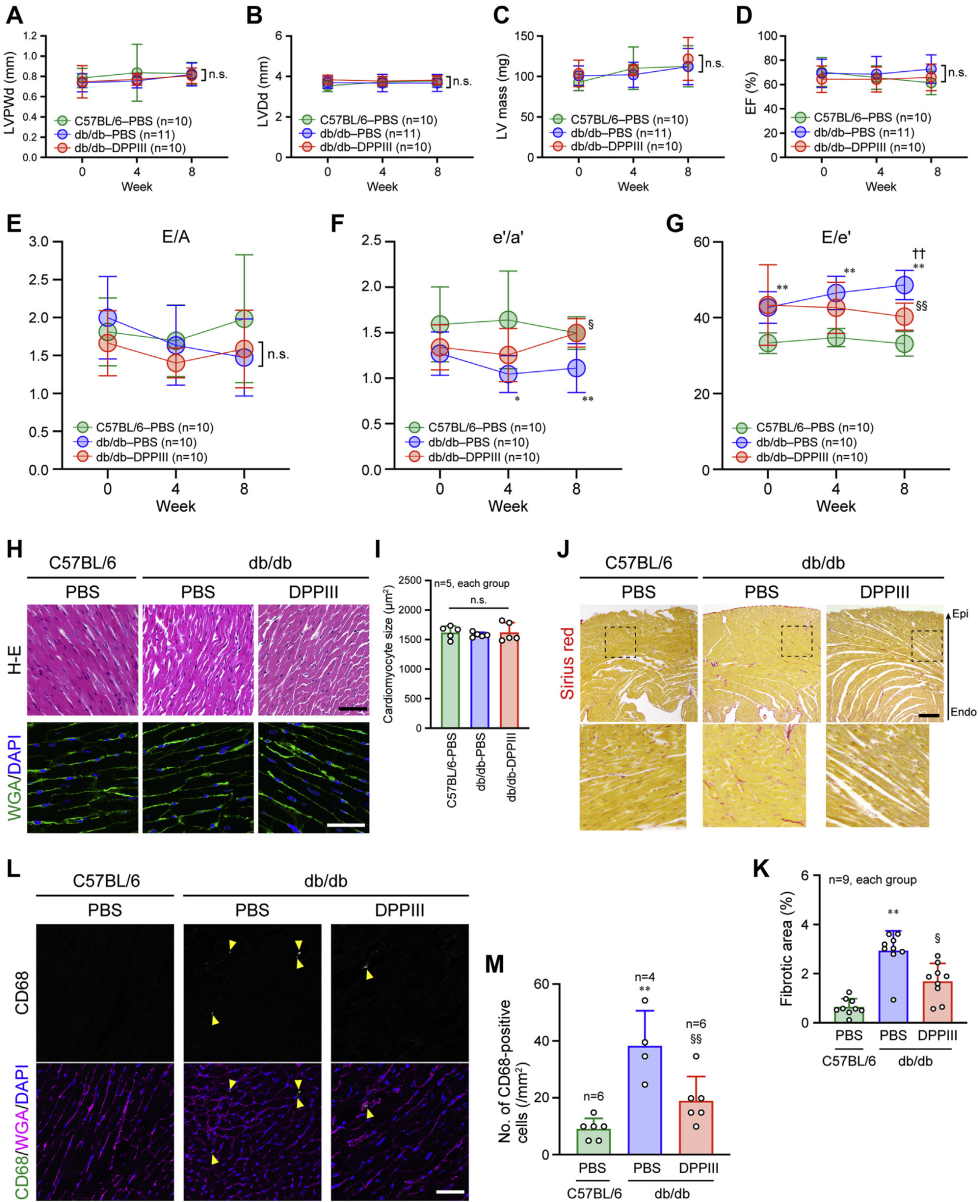
**Cardioprotective effect of DPPIII on diabetic mice.***A*–*G*, echocardiographic measurement of each parameter analyzed every 4 weeks during the 8-weeks experimental period. *H*, hematoxylin-eosin (H-E) and WGA staining of the mouse heart after the 8-week treatment. *I*, summary graph of cardiomyocyte size measured by WGA staining. *J*, cardiac fibrosis detected by Sirius *red* staining after the 8-week treatment. The *dotted square* was enlarged and shown below. *K*, summary graph of the percentage of fibrotic areas in the cardiac sections. *L*, confocal images of CD68 immunostaining. Cell membrane and nuclei were counterstained with WGA and DAPI, respectively. *M*, summary graph of the number of CD68-positive cells. Scale bars: 50 μm (*H* and *L*) and 200 μm (*J*). In *A*–*G*, two-way ANOVA was applied for comparing the data between groups, and one-way ANOVA was applied for comparing the results at week 0 with those at other time points; in *I*, *K*, and *M*, one-way ANOVA was used to compare the data of each group. ∗*p* < 0.05 and ∗∗*p* < 0.01 *versus* C57BL/6 mice; ^††^*p* < 0.01 *versus* 0 weeks; ^§^*p* < 0.05 and ^§§^*p* < 0.01 *versus* PBS-infused db/db mice. e'/a', ratio of early to atrial diastolic mitral annular velocities; E/e', ratio of peak transmitral velocity of early inflow to early diastolic mitral annular velocity; E/A, ratio of peak transmitral velocity of early inflow to atrial inflow; EF, ejection fraction; LVDd, LV diastolic diameter; LVPWd, left ventricular posterior wall diastolic thickness.

Cardiac function during DPPIII administration was monitored by echocardiography. As previously reported (32, 33), we found that db/db mice displayed normal left ventricular (LV) dimensions and systolic functions without hypertrophy, which were similar to C57BL/6 mice, and that the parameters were not significantly changed during the 8-week DPPIII treatment (Fig. 1, *A*–*D*, Fig. S2, and Movies S1–S3). In contrast, the diastolic functions in the heart assessed by pulse-wave and tissue Doppler modes of echocardiography were impaired in PBS-infused db/db mice, compared with PBS-infused C57BL/6 mice (Fig. 1, *E*–*G*, Fig. S2, *A* and *B*, and Movies S1 and S2). Notably, DPPIII treatment improved the impaired diastolic functions in db/db mice (Fig. 1, *E*–*G*, Fig. S2*C*, and Movie S3). Hematoxylin and eosin (H-E) and wheat germ agglutinin (WGA) staining demonstrated that the morphology and size of cardiomyocytes were similar between C57BL/6 and db/db mice and were unchanged by DPPIII treatment (Fig. 1, *H* and *I*), whereas compared with the C57BL/6 mouse heart, the fibrosis was increased in the db/db mouse heart, which was reduced by DPPIII treatment (Fig. 1, *J* and *K*). Cardiac fibrosis is induced by enhanced inflammation with infiltrated monocytes and macrophages into the myocardium (34). We found increased infiltration of CD68-positive inflammatory cells in the PBS-infused db/db than C57BL/6 mouse hearts, which was reversed in the db/db mouse heart when treated with DPPIII (Fig. 1, *L* and *M*). These results suggest that DPPIII prevents DM-related diastolic dysfunction by reducing inflammation and fibrosis in the heart.

### Protective effects of DPPIII on DKD

**Figure 2:**
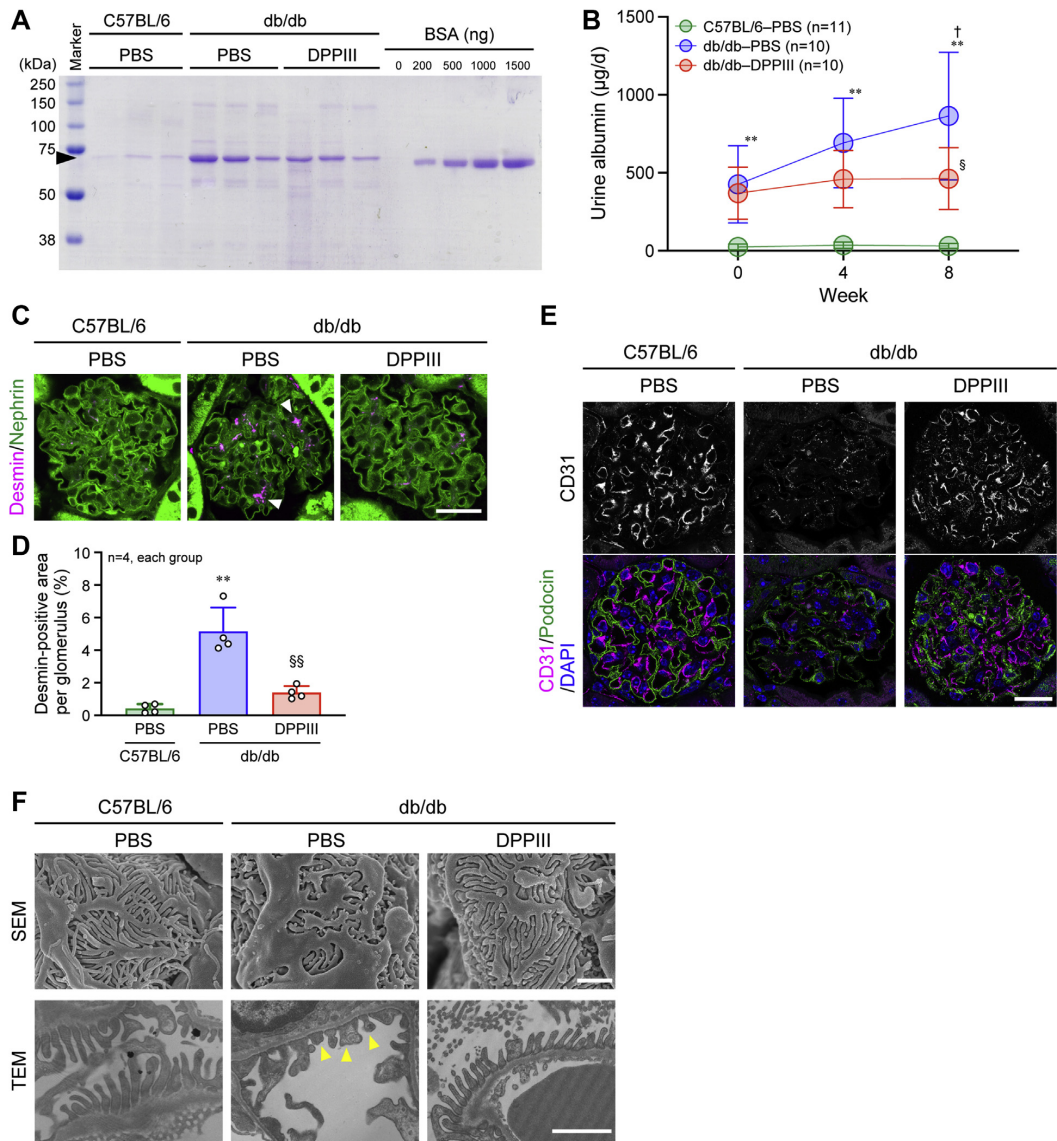
**Reno-protective effect of DPPIII on diabetic mice.***A*, representative Coomassie Brilliant Blue staining of urine samples after 8 weeks and different amounts of bovine serum albumin (BSA). The urine samples from mice were collected for 24 h (1 day), and the proteins in the samples were separated by SDS-PAGE. An *arrowhead* indicates urine albumin. BSA was used as a control. *B*, summary graph of the amount of excreted albumin for 1 day in each group. *C*, confocal images of desmin and nephrin co-immunostaining in the glomeruli. *Arrowheads* indicate abnormal desmin deposition. *D*, summary graph of the percentage of desmin-positive areas per glomerulus. *E*, confocal images of CD31 and podocin co-immunostaining in the glomeruli. *F*, ultrastructures of the glomeruli observed by scanning electron microscopy (SEM) and transmission electron microscopy (TEM). *Arrowheads* indicate damaged podocyte foot processes. Scale bars: 20 μm (*C* and *E*) and 1 μm (*F*). In *B*, two-way ANOVA was applied for comparing the data between groups, and one-way ANOVA was applied for comparing the results at week 0 with those at other time points; in *C*, one-way ANOVA was used to compare the data of each group. ∗∗*p* < 0.01 *versus* C57BL/6 mice; ^†^*p* < 0.05 *versus* 0 weeks; ^§^*p* < 0.05 and ^§§^*p* < 0.01 *versus* PBS-infused db/db mice.

One of the symptoms of DKD is albuminuria (14, 35). Even at the beginning of the experiment, db/db mice exhibited a higher level of urine albumin excretion than C57BL/6 mice, and the excretion was markedly elevated in PBS-infused db/db mice, whereas the increase was blocked by DPPIII treatment (Fig. 2, *A* and *B*). In parallel with the progression of albuminuria, desmin deposition, an indicator for kidney injury, in the glomeruli was promoted in PBS-infused db/db mice, which was almost totally reversed by DPPIII treatment (Fig. 2, *C* and *D*). Furthermore, the glomerular microvasculature detected by CD31-positive endothelial cell staining was disrupted in PBS-infused db/db mice, compared with PBS-infused C57BL/6 mice, and that this disruption was recovered in DPPIII-treated db/db mice (Fig. 2*E*). Observation of ultrastructures of the glomeruli clearly depicted the effacement of podocyte foot processes in PBS-infused db/db mice, and the podocyte damage was ameliorated by DPPIII treatment (Fig. 2*F*). These results suggest that DPPIII functions as a suppressor of DKD.

### Identification of DPPIII-cleaved cytotoxic peptides in db/db mouse plasma

**Figure 3:**
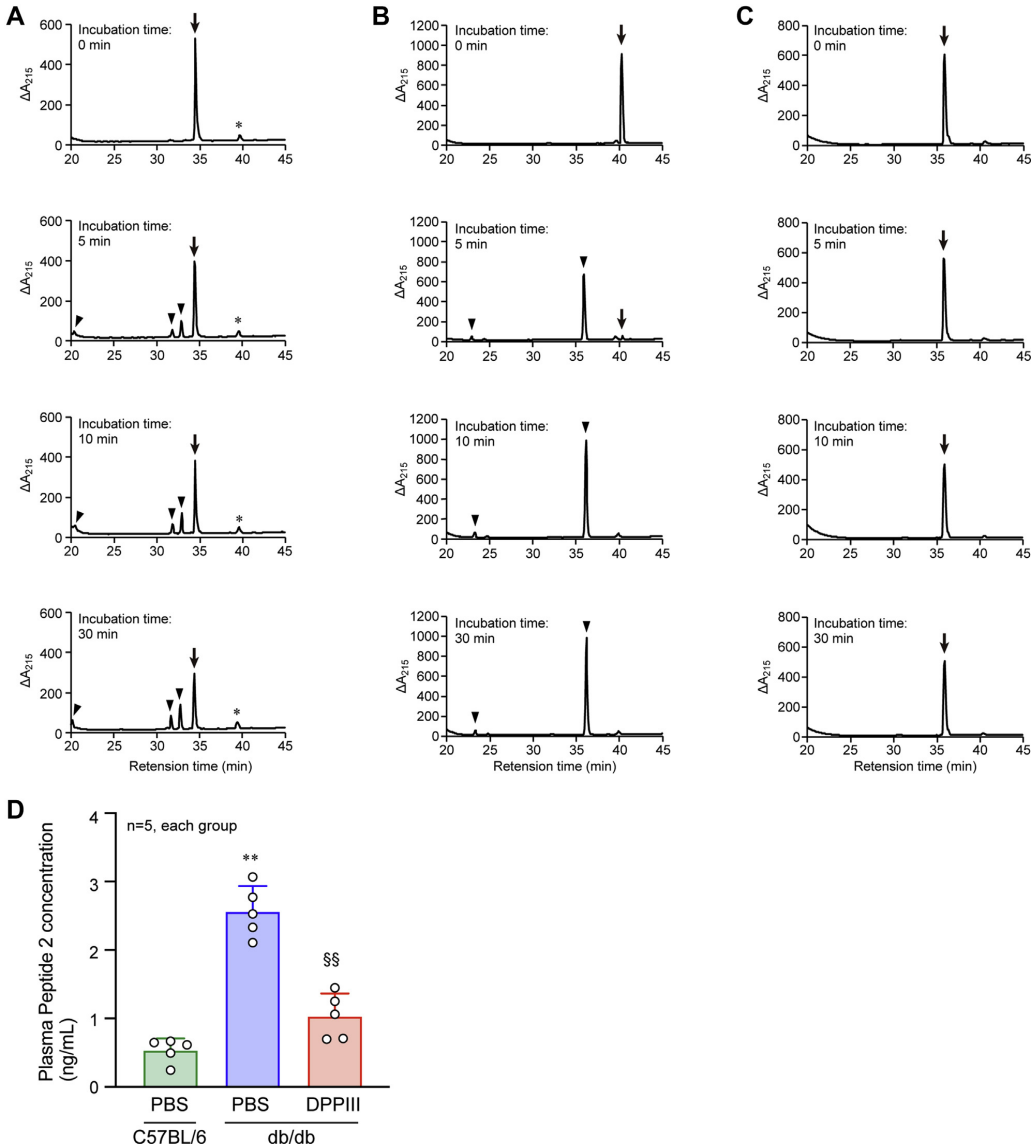
**Proteolytic activity of DPPIII on peptides identified in the plasma of diabetic mice.***A*–*C*, Peptide 1 (*A*), Peptide 2 (*B*), or Peptide 3 (*C*) was incubated with DPPIII for the indicated times, and the cleaved products were analyzed by reversed-phase liquid chromatography. *Arrows*: Peptide 1 (*A*), Peptide 2 (*B*), or Peptide 3 (*C*); *arrowheads*: cleaved products; ∗: nonspecific contaminant. *D*, plasma Peptide 2 concentration in the mice of each group was determined by SRM. One-way ANOVA was used to compare the data of each group. ∗∗*p* < 0.01 *versus* C57BL/6 mice; ^§§^*p* < 0.01 *versus* PBS-infused db/db mice.

Because DPPIII treatment induced cardio- and reno-protection in the diabetic mice independently of the amelioration of hyperglycemia, we hypothesized that DPPIII might effectively cleave harmful cytotoxic peptides that are present in db/db mouse blood. To explore the peptides, the whole peptidomics analysis was conducted using tandem mass spectrometry, in which peptides derived from the plasma of PBS-infused C57BL/6 mice, PBS-infused db/db mice, and DPPIII-treated db/db mice were compared. In the comparison, we focused on the short-length peptides consisting of ≤12 amino acids because of the specificity of DPPIII digestion, and the lists of the peptides in the three mouse groups were shown in Supplemental Data 1 to 3. Then, we used the following strategy to identify the target peptide: (1) selecting the peptides that existed in the PBS-infused db/db mouse plasma, but not in the PBS-infused C57BL/6 mouse plasma, (2) selecting the peptides of which amount (Area) was higher in the PBS-infused db/db mouse plasma than in the PBS-infused C57BL/6 mouse plasma, (3) among the peptides that met the criteria (1) or (2), selecting the peptides of which N-terminal two amino acids were deleted or the peptides that were not detected in the DPPIII-treated db/db mouse plasma, and then, (4) selecting the peptides that have the arginine or tyrosine residue within the N-terminal two amino acids because the peptides cleaved by DPPIII include the residue (28). After performing these procedures, three peptides remained (Table 1). Subsequently, we determined whether DPPIII could cleave these peptides. Each peptide was incubated with DPPIII, and then, the peptide with degraded products was separated by reversed-phase liquid chromatography. Only Peptide 2 was rapidly and completely cleaved by DPPIII, while the other peptides were not or were ineffectively digested (Fig. 3, *A*–*C*). The plasma concentration of Peptide 2 in mice of each group was determined by selected reaction monitoring (SRM). The concentration is significantly higher in the plasma of PBS-infused db/db mice compared with PBS-infused C57BL/6 mice and is clearly reduced by administration of DPPIII in db/db mice (Fig. 3*D*). As shown in Table 1, Peptide 2 is a part of complement component 3 (C3) and consists of nine amino acids, being 53% identical with C3f (17 amino acids), which is generated from C3b. C3f has been reported to have anaphylatoxic biological activity such as C3a (36).

**Table 1:** Short-length peptides that existed in the plasma of PBS-infused db/db mice and had potential to be cleaved by DPPIII

Peptide	Sequence	Original protein name	Accession no.	Position of identified peptide in the original protein
1	RGVSAEYSF	Sorting and assembly machinery component 50 homolog	NP_848729	209–217
2	RLLWENGNL	Complement component 3	NP_033908	1310–1318
3	RPHFLYPKSRLV	Clusterin	NP_038520	214–225

### Cytotoxic role of Peptide 2 in the heart and kidney

We examined the *in vivo* cytotoxicity of Peptide 2 by intravenous injection of this peptide (0.5 μg/g body weight) into mice. As a control peptide, we selected a peptide that also consists of nine amino acids (IAFSQYLQK) derived from serum albumin and was present at the same level in the plasma of both PBS-infused and DPPIII-treated db/db mice in the peptidomics analysis as described above (datasets 2 and 3). The control peptide was not digested by DPPIII (Fig. S3). The body weight and blood glucose level in db/db mice increased similarly during the experimental period with both the control peptide and Peptide 2 treatments (Fig. S4, *A* and *B*). Hemodynamics and plasma lipid profiles were also almost unchanged and similar between the peptide treatments (Fig. S4, *C*–*H*).

**Figure 4:**
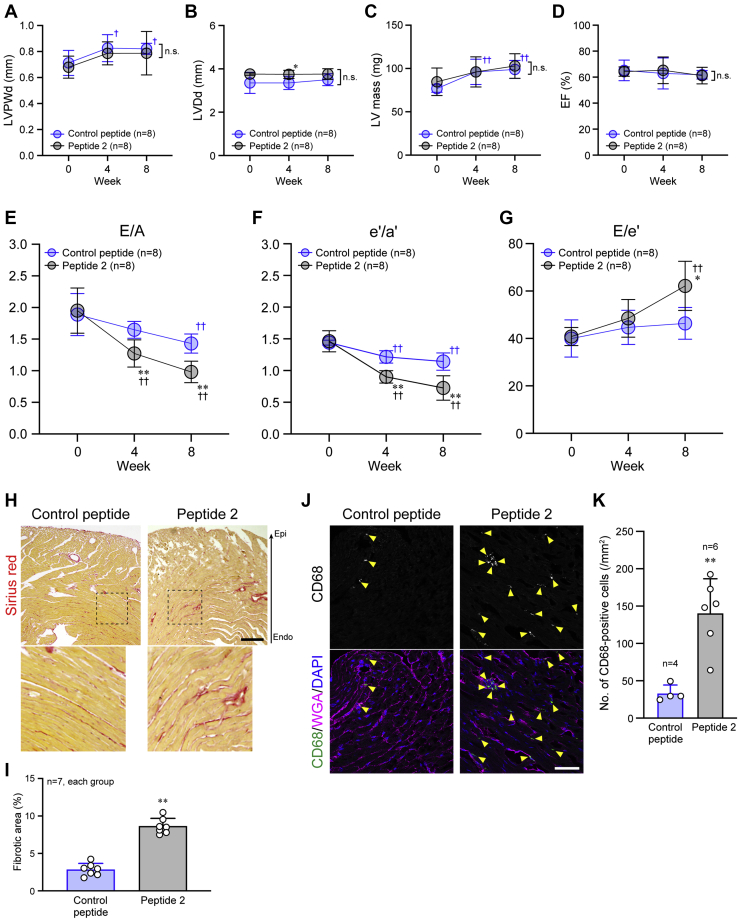
**Deterioration of cardiac diastolic function by Peptide 2 administration.***A*–*G*, echocardiographic measurement of each parameter analyzed every 4 weeks during the 8-week experimental period. *H*, cardiac fibrosis detected by Sirius red staining after the 8-week treatment. The *dotted square* was enlarged and shown below. *I*, summary graph of the percentage of fibrotic areas in the cardiac sections. *J*, confocal images of CD68 immunostaining. Cell membrane and nuclei were counterstained with WGA and DAPI, respectively. *K*, summary graph of the number of CD68-positive cells. Scale bars: 200 μm (*H*) and 50 μm (*J*). In *A*–*G*, two-way ANOVA was applied for comparing the data between groups, and one-way ANOVA was applied for comparing the results at week 0 with those at other time points; in *I* and *K*, the data between the two groups were analyzed by Kolmogorov–Smirnov test. ∗*p* < 0.05 and ∗∗*p* < 0.01 *versus* Control peptide; ^††^*p* < 0.01 *versus* 0 week.

**Figure 5:**
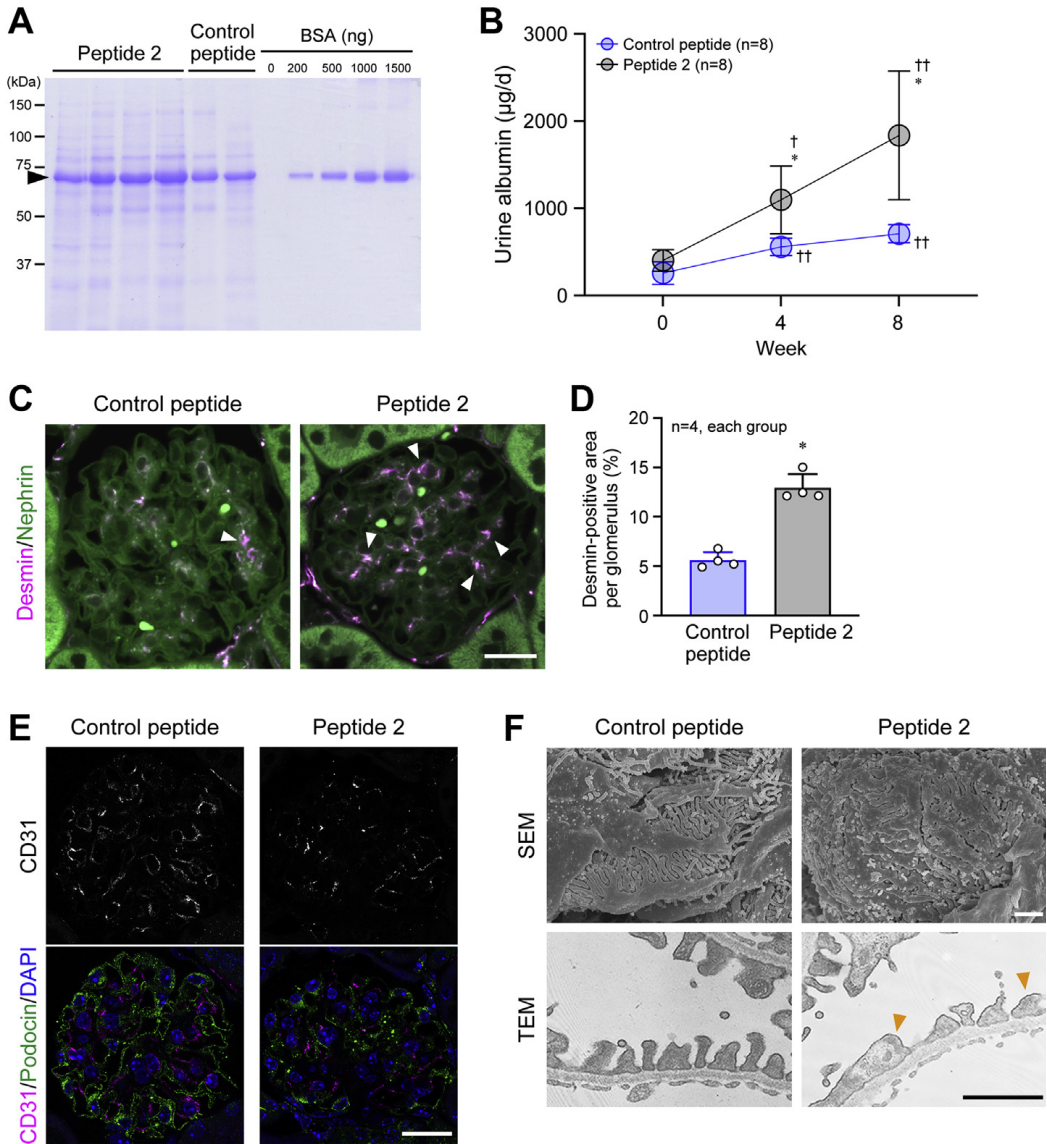
**Peptide-2-mediated deterioration of renal function.***A*, representative Coomassie Brilliant *Blue* staining of urine samples after 8-week treatment and different amounts of BSA. The urine samples from mice were collected for 24 h (1 day), and the proteins in the samples were separated by SDS-PAGE. An *arrowhead* indicates urine albumin. *B*, summary graph of the amount of excreted albumin for 1 day in each group. *C*, confocal images of desmin and nephrin co-immunostaining in the glomeruli. *Arrowheads* indicate abnormal desmin deposition. *D*, summary graph of the percentage of desmin-positive areas per glomerulus. *E*, confocal images of CD31 and podocin co-immunostaining in the glomeruli. *F*, ultrastructures of the glomeruli observed by scanning electron microscopy (SEM) and transmission electron microscopy (TEM). *Arrowheads* indicate highly damaged podocyte foot processes. Scale bar: 20 μm (*C* and *E*) and 1 μm (*F*). In *B*, two-way ANOVA was applied for comparing the data between groups, and one-way ANOVA was applied for comparing the results at week 0 with those at other time points; in *D*, the data between the two groups were analyzed by Kolmogorov–Smirnov test. ∗*p* < 0.05 *versus* Control peptide; ^†^*p* < 0.05 and ^††^*p* < 0.01 *versus* 0 week.

We then investigated the effect of Peptide 2 on the heart of db/db mice. LV dimensions and systolic functions were normal and preserved by Peptide 2 administration (Fig. 4, *A*–*D*, Fig. S5, and Movies S4 and S5). However, cardiac diastolic functions were further deteriorated by Peptide 2 administration, compared with the control peptide administration (Fig. 4, *E*–*G*, Fig. S5, and Movies S4 and S5). Similarly, cardiac fibrosis and inflammatory cell infiltration were enhanced by Peptide 2 administration (Fig. 4, *H*–*K*). Peptide 2 also displayed harmful effects on the kidney of db/db mice. The urine albumin excretion was more abundant by Peptide 2 than the control peptide injection (Fig. 5, *A* and *B*). Desmin deposition and microvasculature disruption in the glomeruli were highly enhanced by Peptide 2 injection, compared with the control peptide injection (Fig. 5, *C*–*E*). In accordance with these results, ultrastructures of the glomeruli demonstrated remarkable Peptide 2-induced damage to podocyte foot processes (Fig. 5*F*). Peptide 2 administration in nondiabetic db/m mice did not affect their body weight, blood glucose level, or hemodynamics (Fig. S6, *A*–*D*). The deteriorative effects of Peptide 2 on the organs in db/db mice were not observed in db/m mice (Figs. S6, *E*–*J*, and S7). These results suggest that Peptide 2, which is a substrate of DPPIII, accelerates DM-related organ damage and dysfunctions, particularly in the heart and kidney, and that the peptide is less harmful in nondiabetic animals.

### Mechanism of Peptide-2-induced cytotoxicity

**Figure 6:**
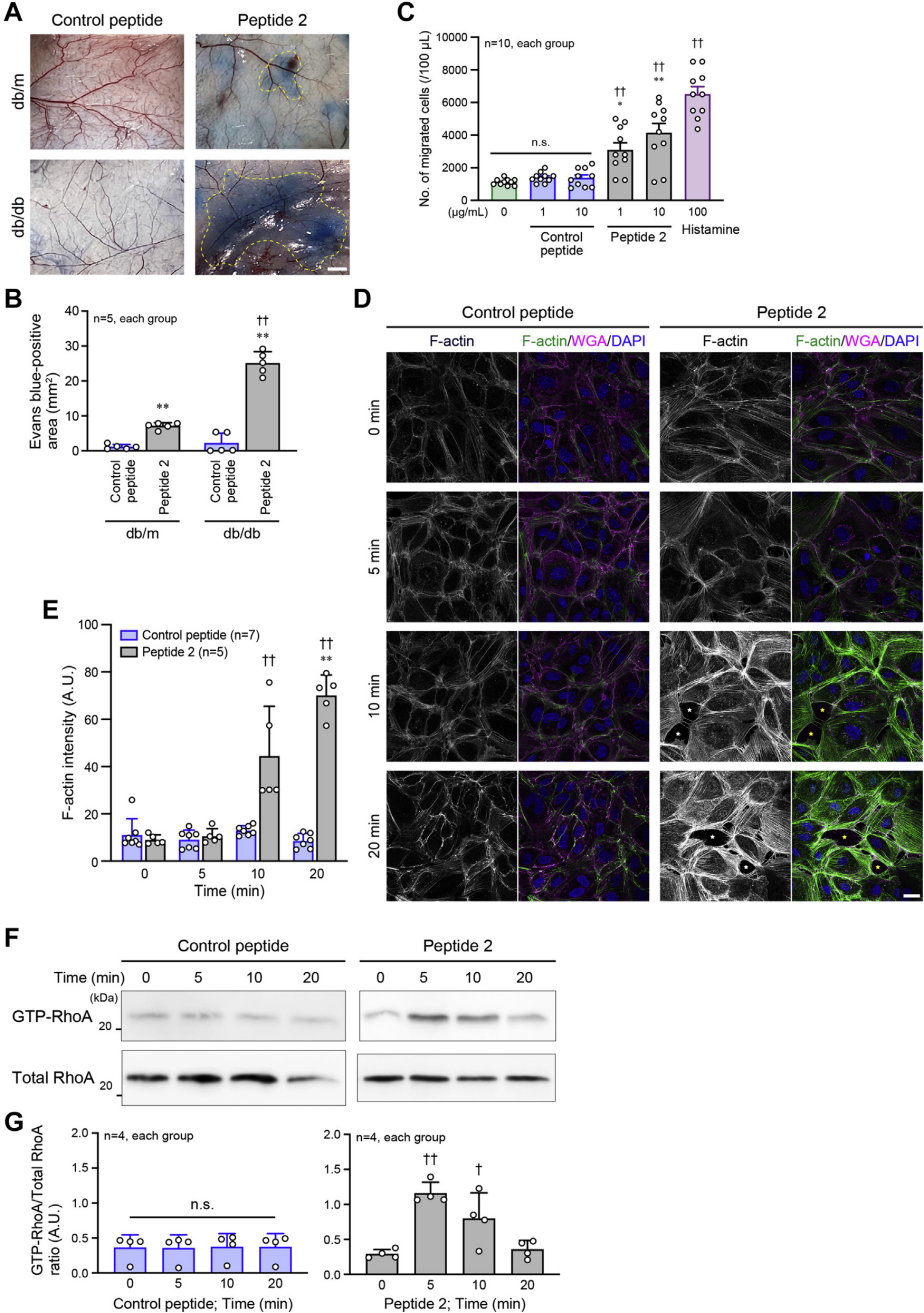
**Peptide-2-induced vascular permeability and RhoA activation in endothelial cells.***A*, miles assay to visualize the vascular permeability. *Dotted areas* indicate the extravasated Evans *blue*. *B*, summary graph of Evans *blue*-positive area. *C*, migration activity of U937 cells seeded on a monolayer of HUVECs in a Transwell chamber. Histamine was used as the positive control. *D*, F-actin assembly in the HUVECs treated with Peptide 2 for the indicated times. F-actin was stained with Alexa488-conjugated phalloidin. Cell membrane was stained with WGA. Stars indicate the gaps between cells. *E*, summary graph of phalloidin intensity examined in (*D*). *F*, RhoA activity in HUVECs treated with Peptide 2 for the indicated times. The active form of RhoA was pulled down using GST fusion protein including GTP-bound RhoA-binding domain of mDia. *G*, summary graph of RhoA activity examined in (*F*). Scale bars: 2 mm (*A*) and 20 μm (*D*). In *B* and *E*, two-way ANOVA was applied for comparing the data between groups; in *C*, *E* and *G*, one-way ANOVA was used for comparing the results at 0 μg/ml or Time 0 with those at other concentrations or time points. ∗*p* < 0.05 and ∗∗*p* < 0.01 *versus* Control peptide; ^†^*p* < 0.05 and ^††^*p* < 0.01 *versus* 0 μg/ml, db/m or 0 min. A.U., arbitrary units.

To investigate how Peptide 2 induces toxicity in the heart and kidney, we hypothesized that this peptide had anaphylatoxin-like functions because of its representing a part of C3f. The Miles assay demonstrated the marked induction of vascular permeability on stimulation with Peptide 2 as well as with histamine, a positive control, but not on control peptide injection (Fig. 6*A* and *B*). Subsequently, chemoattractant activity of Peptide 2 was examined using U937 macrophage-like cells that were seeded onto a monolayer of human umbilical vein endothelial cells (HUVECs) in the Transwell assay. Peptide 2 stimulation in the lower chamber increased the number of migrated U937 cells across the monolayer in a concentration-dependent manner (Fig. 6*C*). We found that Peptide 2 stimulation clearly promoted F-actin assembly in HUVECs, compared with control peptide stimulation (Fig. 6, *D* and *E*). Excessive F-actin assembly inside cells causes strong contractility and high inward traction forces from the plasma membrane (37, 38), physically inducing the breakdown of intercellular junctions. Consistent with this, several gaps between HUVECs were observed by Peptide 2 stimulation (Fig. 6*D*), thereby allowing transendothelial migration of the U937 cells. These phenomena disappeared when peptides derived from DPPIII-digested Peptide 2 were used (Fig. S8), suggesting the specific contribution of Peptide 2, but not its degraded peptides, to the phenomena. RhoA activity plays a role in F-actin assembly (39), and Peptide 2 stimulation significantly increased the GTP-bound active RhoA in HUVECs, whereas the control peptide stimulation had no effect (Fig. 6, *F* and *G*; Fig. S9).

**Figure 7:**
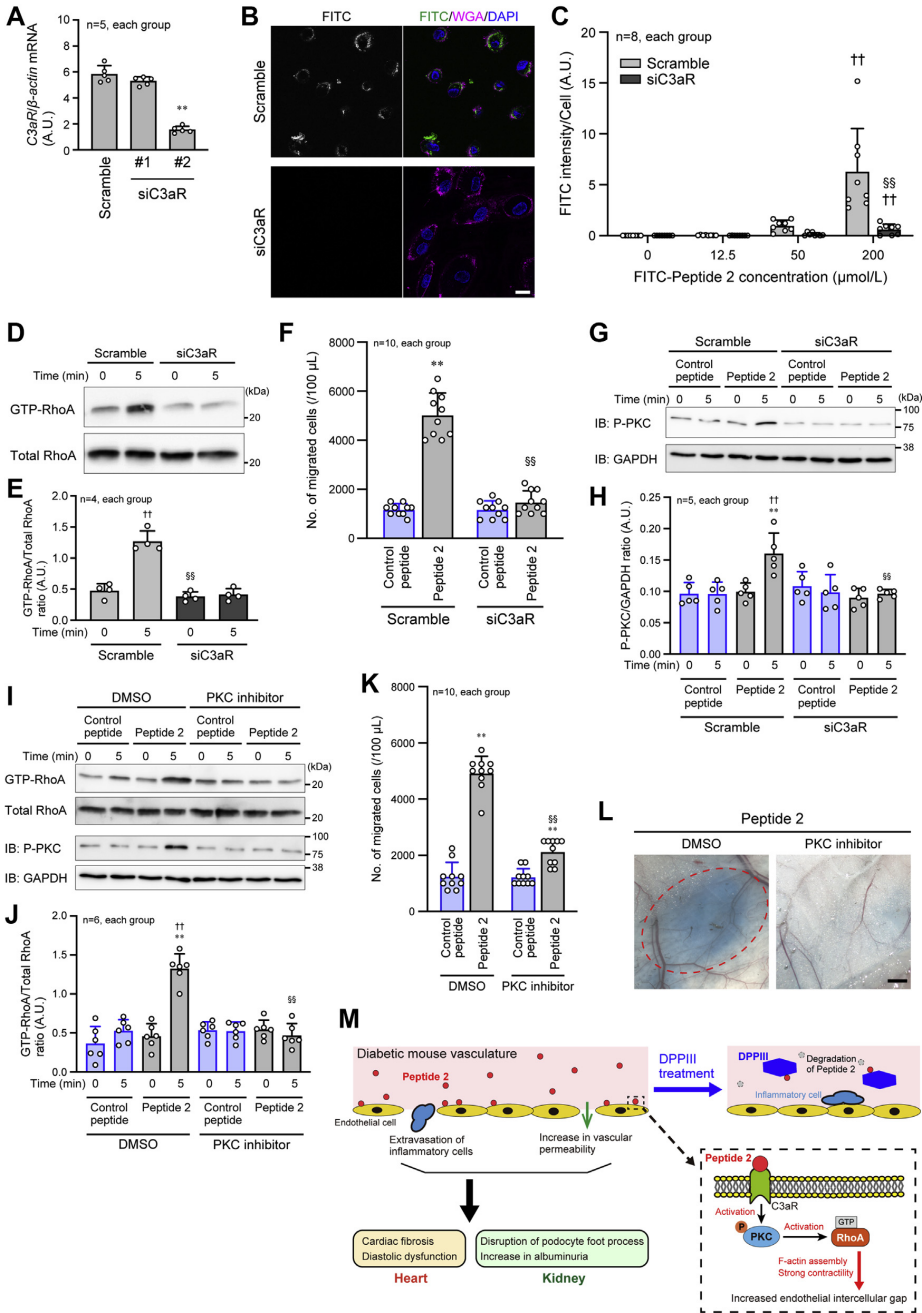
**Involvement of C3aR and protein kinase C in Peptide-2-induced activation of RhoA and vascular permeability.***A*, semiquantitative PCR to monitor the knockdown efficiency of C3a receptor (C3aR) in HUVECs by siRNA against C3aR (siC3aR). *B*, FITC-Peptide 2 attachment on HUVECs observed by fluorescent confocal microscopy. HUVECs were incubated with 200 μmol/l FITC-Peptide 2. *C*, summary graph of the intensity of FITC-Peptide 2 on HUVECs, which were incubated with the indicated concentrations of FITC-Peptide 2. *D*, inhibition of Peptide-2-induced RhoA activation by knockdown of C3aR in HUVECs. HUVECs were incubated with Peptide 2 for the indicated times. *E*, summary graph of RhoA activity examined in (*D*). *F*, inhibition of Peptide-2-induced U937 cell migration through a HUVEC monolayer coating in the Transwell chamber by knockdown of C3aR in HUVECs. *G*, protein kinase C (PKC) phosphorylation (activation) induced by Peptide 2 with or without siC3aR in HUVECs. *H*, summary graph of the P-PKC/GAPDH density ratio. *I*, RhoA activity induced by Peptide 2 in the presence and absence of PKC inhibitor in HUVECs. *J*, summary graph of RhoA activity examined in (*I*). *K*, Peptide 2-induced U937 cell migration in the Transwell chamber coated with HUVECs in the presence and absence of PKC inhibitor. *L*, Peptide-2-stimulated vascular permeability in the presence and absence of PKC inhibitor. *Dashed oval* indicates the extravasated Evans *blue*. *M*, schematic representation of the protective effect of DPPIII on the heart and kidney. Scale bar: 20 μm (*B*) and 1 mm (*L*). In *A*, the data was analyzed by one-way ANOVA; in *C*, *H* and *J*, two-way ANOVA was applied for comparing the data between groups, and one-way ANOVA was applied for comparing the results at 0 μmol/l or Time 0 with those at other concentrations or time points; in *F* and *K*, two-way ANOVA was used to compare the data between groups. ∗∗ *p* < 0.01 *versus* Control peptide; ^†^*p* < 0.05 and ^††^*p* < 0.01 *versus* 0 min; ^§§^*p* < 0.01 *versus* Scramble or DMSO. A.U., arbitrary units; P-PKC, phosphorylated PKC.

We further examined how Peptide 2 transduced the signal inside the endothelial cells to induce RhoA activation and F-actin assembly by focusing on the association of Peptide 2 with C3a receptor (C3aR), because C3f is considered to exert its anaphylatoxic activity through C3aR (36). Following confirmation of C3aR knockdown in HUVECs by siRNA (siC3aR #2) (Fig. 7*A*), fluorescein isothiocyanate (FITC)-conjugated Peptide 2 was incubated with HUVECs. FITC-Peptide 2 attachment on the cells detected by fluorescence microscopy was significantly reduced in C3aR-knockdown HUVECs, compared with scramble RNA-transfected HUVECs (Fig. 7, *B* and *C*). This suggests Peptide 2 binding to C3aR. RhoA activation and U937 transendothelial migration induced by Peptide 2 were almost completely inhibited by C3aR knockdown (Fig. 7, *D*–*F*; Fig. S9). Because protein kinase C (PKC) functions downstream of C3aR (40, 41) and mediates the activation signal for RhoA (42), the involvement of PKC in the Peptide-2-related cytotoxic activity was investigated in HUVECs. Peptide 2 stimulation increased PKC phosphorylation, and the phosphorylation was diminished by C3aR knockdown (Fig. 7, *G* and *H*; Fig. S9). Consequently, Peptide-2-induced RhoA activation and U937 transendothelial migration were inhibited in the presence of PKC inhibitor (Fig. 7, *I*–*K*; Fig. S9). We also found that in the Miles assay, Peptide-2-stimulated vascular permeability was inhibited by PKC inhibitor (Fig. 7*L*). These results suggest that Peptide 2 utilizes the C3aR-PKC signaling pathway to induce cytotoxicity, leading to DM-mediated organ injury and failure.

## Discussion

DM patients have a high mortality rate, mainly due to DM-related heart and/or kidney failure (35, 43). Pathologically, heart dysfunction exacerbates kidney damage, while renal injury negatively affects cardiac function. This is clinically referred to as cardiorenal syndrome, which displays mutual deterioration between the heart and kidney and contributes to the increased mortality (44). To reduce diabetic complications in tissues and organs, particularly in the heart and kidney, great effort has been made to develop effective medicines and therapeutics for DM. Among these, much attention has recently been paid to sodium-glucose cotransporter 2 (SGLT2) inhibitors that lower blood glucose and HbA1c levels by blocking glucose reabsorption from proximal tubules. Treatment with canagliflozin, an SGLT2 inhibitor, for DM patients improves heart failure and DKD (45, 46). It is clear that improving hyperglycemia contributes to decreased morbidity and mortality of DM. However, there are patients with controlled blood glucose who still suffer from DM-related complications. Therefore, it is also necessary to develop blood-glucose-independent therapies that limit DM-induced cardiac and renal injuries.

We demonstrated in this study that DPPIII has cardio- and reno-protective effects in a diabetic mouse model. DPPIII treatment in these mice did not alter the blood glucose level, suggesting that it has a blood-glucose-independent mechanism. We proposed the beneficial function of intravenously administered DPPIII in this and previous studies (30), whereas endogenous DPPIII has no signal sequence and principally present in the cytosol, probably contributing to intracellular protein catabolism (47). Extending these findings, DPPIII was shown to have important nonenzymatic functions inside cells (48). DPPIII regulates the Keap-1/Nrf-2 signaling pathway by binding to Keap-1. When DPPIII binds to Keap-1, Nrf-2, a transcription factor, is released from Keap-1 and is resistant to ubiquitination. This promotes Nrf-2 translocation from the cytosol into the nucleus to induce the gene expression of antioxidant enzymes and other cytoprotective enzymes against oxidative stress (49, 50).

In addition to DPPIII in the cells, extracellular DPPIII has been observed and its significance is currently being investigated. In the process of antigen presentation, DPPIII leaking from necrotic cells digests and eliminates oligopeptides necessary for cross-priming and activation of naïve CD8^+^ T cells, preventing necrotic-cell-dependent immunogenicity and autoimmune disorders (51). Recently, it is shown in severely burned patients that plasma DPPIII discharged from damaged cells may be a biomarker for an increased risk for mortality, circulatory failure, and acute kidney injury (52). It is also reported that while the mechanism by which DPPIII is excreted from the cytosol remains unclear, the plasma DPPIII concentration in patients with heart failure is associated with mortality (53). DPPIII may act as a myocardial depressant, because DPPIII inhibition by the monoclonal antibody Procizumab improves cardiac function in the heart failure rodent models (53, 54). There are some methodological discrepancies between these and our studies. First, in the recent studies from another research group, the protective effect of Procizumab on the heart was only examined for the short duration (24 h), which was different from our present study that analyzed the cardiac and renal functions for relatively long period (8 weeks) with the repeated administration of DPPIII into mice. The long-term effect of Procizumab has not been certified yet. Second, the circulatory and cardiac conditions in the recent experimental studies were apart from ours. In contrast to our study, cardiac systolic function was significantly impaired by isoproterenol injection and sever hypotension was induced by the septic stimulation, before treatment with Procizumab was conducted in the recent studies. These discrepancies may explain the inconsistency in the conclusion of the recent and our studies concerning the role of DPPIII in the cardiac and renal functions.

Moreover, in the recent studies, the mechanism by which plasma DPPIII affects mortality and cardiac function was not clearly presented. One of the DPPIII substrates in the plasma is AngII, and the inhibitory effect of Procizumab on DPPIII may result in the increase in AngII. If this is the case, it might be inconsistent, because an excess of plasma AngII is commonly accepted to be deteriorative for many organs including the heart. There is a clear consensus that appropriate blockade of the renin–AngII–aldosterone system is clinically beneficial for the treatment of heart and/or kidney failure (55, 56). It remains unclear in the above studies how the very low plasma DPPIII level (pmol/l level) exerts strong bioactivities in the entire body, and thus, further investigations are needed to address these issues in the future.

We newly identified the peptide, Peptide 2, which was cleaved by DPPIII and was a substrate of DPPIII in the plasma of diabetic mice. The peptide exacerbated diabetic dysfunctions in the heart and kidney in the db/db mice, but not in the nondiabetic db/m mice. It is well known that DM (hyperglycemia) preferentially causes endothelial dysfunctions and microvascular damages. Thus, vessels in diabetic animals are fragile for additional stimulation and/or stress to induce vascular permeability, facilitating the initiation and progression of complications in the organs (57, 58, 59). This may explain the different pathological phenomena observed in the heart and kidney between db/db and db/m mice, in response to intravenous Peptide 2 administration.

Peptide 2 is a part of C3f (53% identical), and C3f can act as an anaphylatoxin such as C3a (36). Peptide 2 transduced intracellular signaling toward anaphylatoxin-like activity by interacting with C3aR in HUVECs. In endothelial cells, C3aR couples to G_α12_ or G_α13_, and the receptor activation induces actin stress fiber formation through the Rho signal cascade downstream of the G proteins (60). This may impair the endothelial function and facilitate abnormal extravasation of inflammatory cells. Recent findings show that loss or blockade of C3aR improves renal dysfunction. C3aR knockout mice develop less severe renal damage than wild-type mice in the diabetic model induced by streptozotocin administration and high-fat diet (61). Treatment with a C3aR antagonist in diabetic mice limits glomerular injury and proteinuria by preserving the podocyte density (62). Therefore, inhibition of C3aR activation may play a protective role in DM-induced organ damage.

C3 is the central element in the complement system that is pivotal for innate immunity (63). C3-derived molecules, such as C3a, C3b, and C3f, have diverse bioactivities, and their uncontrolled overactivation induces injury in tissues and organs. In contrast, C3 deficiency significantly reduces renal interstitial fibrosis by inhibiting inflammatory responses in the mouse ureteral obstruction model (64). C3 is cleaved by C3 convertase and is divided into two fragments: C3a, a smaller fragment and C3b, a larger fragment. Increased C3a level in patients with chronic heart failure is clinically associated with more frequent cardiovascular events and higher morbidity and mortality and is a biomarker for acute-phase reaction, inflammation, and cellular stress response (65). C3b functions as an alternative pathway convertase by interacting with activated factor B (Bb). When the conversion of C3b to its inactive form iC3b is suppressed, increased and overactivated C3b may potentially confer harmful effects to organs (63). C3f is generated on the cleavage of C3b by the function of factors H and I. To date, the pathophysiological role of C3f has not been extensively investigated except that a previous report demonstrated its C3a-like anaphylatoxic activity by binding to C3aR (36). As described above, our newly identified peptide, Peptide 2, can also exert anaphylatoxic activity, such as hyperpermeability of microvessels in diabetic mice, through C3aR. Because excessive activation of C3-derived molecules and their associating molecules is causal for several renal diseases, their inhibitors, such as a C3-targeting peptide and a factor B-blocking compound, have been developed and are currently under clinical evaluation (66). Accordingly, DPPIII, which directly digests Peptide 2, may become a candidate to prevent the progression of kidney and heart diseases.

Our study has some limitations. One of them is that the results shown in this study are obtained from the diabetic mouse model. To extend the results in this study for the translational research, the safety of purified DPPIII for its intravenous injection into human subjects should be carefully and strictly certified. Because DPPIII itself exists in cells in a variety of animals including human, it might not be difficult to find its safety for future human use. Another unanswered issue is that the enzyme to produce Peptide 2 by digesting from C3f is not identified. It is also unclear what factor is mainly involved in the increase in Peptide 2 in DM: the enhanced activity/expression of Peptide-2-producing enzyme(s) and/or the increased generation of Peptide 2 precursors C3f and C3b. Nonetheless, the strong points of this study are as follows: we could propose the beneficial effect of DPPIII on DM-induced cardiac and renal dysfunction in a glycemic control-independent manner. Further, we successfully identified Peptide 2, a substrate of DPPIII, which was increased in diabetic mice, and showed the cytotoxicity of Peptide 2 to the heart and kidney with its functional mechanism. These might be useful to develop the novel therapies against DM-related complications.

In conclusion, our findings represent the beneficial effect of DPPIII on DM-related cardiac and renal dysfunctions. The effect is, at least in part, dependent on DPPIII-induced cleavage of the plasma peptide, Peptide 2, which causes anaphylatoxic activity through C3aR. The signaling mechanism transduced by Peptide 2 includes PKC and RhoA downstream of C3aR in endothelial cells, resulting in increased permeability of blood vessels and damage of organs, particularly in the heart and kidney. These are schematically summarized in Figure 7*M*. Based on these results, although more extensive experiments are required regarding translational research, DPPIII might have potential for future clinical use in the prevention of DM-induced cardiac and renal failures.

## Experimental procedures

### Cell culture and mice

HUVECs were purchased from Lonza and maintained in Endothelial Cell Growth Medium MV 2 Kit (Takara Bio Inc). U937 cells were purchased from American Tissue Culture Collection and maintained in RPMI-1640 medium including 10% fetal bovine serum supplemented with 2 mmol/l glutamine and an antibiotics cocktail (Nacalai Tesque). We used 8-week-old male C57BL/6, nondiabetic (db/m; BKS.Cg-*m+*/*+Lepr*^*db*^/Jcl) and diabetic (db/db; BKS.Cg-*+Lepr*^*db*^/*+Lepr*^*db*^/Jcl) mice, which were purchased from CLEA Japan Inc. DPPIII (10 μg/g body weight) or PBS was intravenously injected into mice through the tail vein three times weekly for 8 weeks as previously described (30). Mice were housed in specific pathogen-free conditions in the Research Center for Animal Life Science of Shiga University of Medical Science. The animal experiments conducted in this study were approved by Shiga University of Medical Science Animal Care and Use Committee and were performed in accordance with relevant guidelines and regulations, including Animal Research Reporting of *In Vivo* Experiments (ARRIVE) guidelines.

### Blood pressure measurement and echocardiography

Heart rate and arterial BP were measured noninvasively every 2 weeks during the experiment by the plethysmographic tail-cuff method (machine model BP-98-AL, Softron) in conscious mice, as described previously (30). Transthoracic echocardiography was conducted every 4 weeks during the experiment using the Vevo 2100 system (Fujifilm VisualSonics Inc) in anesthetized mice as described previously with some additional procedures (67). The procedure of anesthesia in mice during echocardiography was as follows: the mice were initially anesthetized with 2% isoflurane mixed with 2 l/min air in the induction chamber. Then, the mice were placed on the heating pad in the supine position, and the snout was put within the nose cone connected to the anesthesia system to maintain the anesthesia with 0.5% to 1.5% isoflurane mixed with 1 l/min air. The physical condition of mice during the echocardiography under anesthesia was checked by heart rate monitoring (Table S1). Cardiac diastolic function was evaluated by several parameters that were monitored by pulse-wave Doppler and tissue Doppler modes. Peak early diastolic (E) and atrial (A) transmitral inflow velocities were obtained by pulse-wave Doppler, and early (e’) and late (atrial: a’) diastolic mitral annular velocities were estimated by tissue Doppler imaging.

### Analyses of blood and urine samples

Blood glucose concentration in mice was measured using the Glutest sensor (Sanwa Kagaku). Plasma cholesterol and triglyceride concentrations were analyzed using the E-test WAKO kit (Fujifilm Wako Pure Chemical Corporation). Mouse urine was collected for 24 h by metabolic cages every 4 weeks during the experiment. The urine samples were analyzed by sodium dodecyl sulfate–polyacrylamide gel electrophoresis (SDS-PAGE), followed by Coomassie Brilliant Blue staining to detect proteins including urinal albumin. The visualized albumin was quantified using ImageJ software.

### Histological staining and immunohistochemistry

Following euthanasia of the mice by cervical dislocation, they were perfused with PBS, and the heart and kidney were extracted. The extracted organs were snap-frozen in liquid nitrogen within a block of water-soluble medium (Surgipath FSC 22, Leica Biosystems) or fixed with 4% paraformaldehyde and subsequently embedded in paraffin blocks. Regarding the frozen samples, they were sectioned in 10 μm thick using a cryostat (Leica Biosystems), fixed with 4% paraformaldehyde on top of poly-L-lysine-coated slides, permeabilized with 0.1% Triton X-100, and blocked with 1% bovine serum albumin (BSA). Primary antibodies (Abs) (see below) were applied in the BSA blocking solution at 4 °C overnight, followed by 1 h incubation at room temperature with fluorescent dye-labeled secondary Abs. Paraffin sections, 4 μm thick, were stained with H-E, WGA (1:500 dilution) (Thermo Fisher Scientific) or Sirius red (1:1000 dilution) (Fujifilm Wako Pure Chemical Corporation) using standard techniques. Confocal images were taken using a Leica SP8 microscope (Leica Microsystems). Images for histological analysis were captured by the BZ-X800 microscope system (Keyence). As described previously (68), at least three random fields were selected from the four sections in each heart to quantify the extent of fibrotic or immunopositive area using ImageJ software.

### Staining of cultured cells

Cells grown on fibronectin-coated glass coverslips were fixed with 4% paraformaldehyde and permeabilized with 0.2% Triton X-100 for 5 min. To visualize F-actin and cell membrane, Alexa Fluor 488-conjugated phalloidin and tetramethylrhodamine-conjugated WGA (1:500 dilution) were applied for 1 h at room temperature in the dark. Following washing of the samples with PBS, nuclei were stained with DAPI (1:500 dilution) (Dojindo) for 5 min.

### Electron microscopy

The mouse kidneys were perfused with 10% formalin in 9 mmol/l sodium cacodylate, 0.05% picric acid, and 135 mmol/l sucrose, and fixed in 4% paraformaldehyde and 2.5% glutaraldehyde in PBS on ice for 2 h. For scanning electron microscopy, the samples were treated with 2% OsO_4_ in PBS for 2 h, dehydrated in a graded series of ethanol (from 60% to 100%) at room temperature, and soaked into 3-methylbutylacetate to be dried completely in Critical Point Dryer (Hitachi). The dried specimens were mounted on brass stages and coated with ionized gold particles (20 nm) in Ion Corter IB-3 (Eiko Engineering). The samples were observed in a JSM-7505FA electron microscope (JOEL Ltd) at an accelerating voltage of 10 kV. For transmission electron microscopy, the samples were washed with PBS, treated with 2% OsO_4_ in PBS on ice for 2 h, dehydrated in graded ethanol, and embedded in epoxy resin. Ultrathin sections were stained with uranyl acetate for 2 h and lead nitrate for 3 min. The samples were observed with a Hitachi H-7500 electron microscope (Hitachi High-Tech).

### Experimental design for peptidomics analysis

Following an 8-week DPPIII or PBS administration, 160 U heparin was intravenously injected in the mice, and blood samples were collected in a tube in which ethylenediaminetetraacetic acid, heparin, and phenylmethylsulfonyl fluoride (PMSF) were added to prevent blood coagulation and protein degradation. The plasma was obtained by centrifugation at 1000*g* at 4 °C for 15 min and applied to reverse-phase chromatography, followed by tandem mass spectrometry. In detail, each plasma sample was diluted with an equal volume of 32% (v/v) acetic acid to favor disruption of peptide/protein interactions and ultrafiltrated with a 10 kDa cutoff filter (Centricon 10, Millipore Corp) to remove the high-molecular-weight proteins. The filtrate was then precipitated with two volumes of cold acetonitrile containing 0.1% of trifluoroacetic acid to complete the removal of any residual high-molecular-weight components. The supernatant from each sample was then dried by Speed Vacuum (Thermo Fisher Scientific), dissolved in 1% (v/v) formic acid. Five microliters of the sample was applied with Nanoflow UPLC (Easy-nLC1000, Thermo Fisher Scientific) for performing liquid chromatography equipped with Nanocolumn (100 μm×10 cm in-house made column packed with a reversed-phase ReproSil-Pur C18-AQ resin [3 μm, 12 nm, Dr Maisch GmbH]). The absorbed peptides were eluted for 100 min with a linear gradient of 0.1% formic acid in acetonitrile (solution B) against 0.1% formic acid in water (solution A).

Subsequently, tandem mass spectrometry analyses were carried out using Orbitrap Q Exactive HF mass spectrometry (Thermo Fisher Scientific) with a spray voltage set to 2.2 kV and ion transfer capillary set to 270 °C. MS1 spectra were collected in the range of 300 to 1650 m/z at 60,000 resolution to hit an AGC target of 3 × 10^6^. The top 20 precursor ions with charge states of 2+ to 6+ were selected for fragmentation with a normalized collision energy of 28%. MS2 spectra were collected at 15,000 resolution to hit an AGC target of 1 × 10^5^. The dynamic exclusion time was set to 30 s. The raw data processing and analysis by database searches were performed with PEAKS STUDIO Desktop Version X referring 36,133 of protein entries in the mouse UniProt database (downloaded on November 6, 2020). The parameters of the software were set as follows: oxidation (+15.99 Da) (histidine and tryptophan residues) and deamidation (+0.98 Da) (asparagine and glutamine residues) were set as considered variable modifications of proteins; nonspecific cleavages were permitted; the precursor ion mass tolerance was set to ±10.0 ppm, and MS/MS tolerance was set to 0.05 Da; threshold score (Expectation value) for accepting individual spectra was set at 15 (−10logP >15); false discovery rate (FDR) estimation was set at 0%. To estimate FDR in the PEAKS studio software, decoy fusion method was applied. This peptidomics analysis was conducted by Creative Dynamics, Inc.

### Proteolytic activity assay

To determine whether DPPIII can cleave candidate peptides detected by the peptidomics analysis, we employed a chromatographic technique as described previously (30). Candidate peptides (Peptides 1–3) and a control peptide, a degradative product of serum albumin, were generated by Biologica Co. Each candidate peptide was dissolved in 200 μl of 50 mmol/l sodium phosphate buffer (pH 8.0) to adjust the final concentration to 50 μmol/l and was incubated with 25 nmol/l DPPIII at 37 °C. The samples were analyzed by reversed-phase chromatographic separation on a COSMOSIL 5C18-AR-300 column (Nacalai Tesque). Chromatograms were processed using Microsoft Excel software.

### Experimental design for SRM

As an internal peptide standard, stable isotope-labeled Peptide 2 was artificially synthesized by Scrum Inc for the mass measurement accuracy. The synthesized Peptide 2 was labeled with [^13^C_6_, ^15^N] leucine at the C-terminal residue. For preparation of plasma samples to apply to liquid chromatography, 10 μl of 1% formic acid in water/acetonitrile (1:1), 10 μl of the isotope-labeled Peptide 2, and 20 μl of 8 mol/l ammonium formic acid were added to 50 μl of the mouse plasma sample, which was then vigorously mixed. Subsequently, 150 μl of methanol/acetonitrile (1:1) was supplied into the sample. High-molecular-weight proteins were removed by centrifugation at 14,000*g* at 4 °C for 5 min. The supernatant from the plasma was then dried by nitrogen gas spraying and dissolved in 1% formic acid in acetonitrile/water (1:9). Three microliters of the sample was applied to liquid chromatography.

SRM experiments were conducted at Kyushu Pro Search LLC using QTRAP6500 (Sciex) coupled to ACQUITY UPLC H-Class-liquid chromatograph (Waters). Each prepared sample was loaded to the ACQUITY UPLC Peptide CSH C18 column (Waters; 13 nm, 1.7 μm, 2.1 mm × 50 mm) at the flow rate of 0.4 ml/min at 50 °C. The gradient elution program with mobile phase A (0.02% (v/v) acetic acid in water) and mobile phase B (0.02% (v/v) acetic acid in acetonitrile) was performed for the determination of Peptide 2; 10 to 30% B for 0 to 3 min, 30% for 3 to 3.5 min, 10% for 3.5 to 4.5 min, 10 to 90% for 4.5 to 5 min, 90% for 5 to 6 min, 10% for 6 to 7 min, 10 to 90% for 7 to 7.5 min, 90% for 7.5 to 8.5 min, and 10% for 8.5 to 10 min. The autosampler was maintained at 10 °C, and the effluent was introduced into an electrospray ionization source operated with positive ion mode. The MS parameters in detail were as follows: curtain gas at 25 psi, collision gas at 12 psi, IonSpray voltage at 5500 V, source temperature at 450 °C, ion source gas 1 at 50 psi, ion source gas 2 at 80 psi. The SRM parameters in details were as follows: declustering potential at 60 V, entrance potential at 10 V, exit potential at 20 V, collision energy at 42 V, and dwell time at 10 ms. SRM of the protonated precursor molecular ions [M + 2H]^2+^ and the related product was quantified. The resolutions of quadrupole Q1 mass (precursor ion) and Q3 mass (product ion) were set at unit. For the SRM scan of Peptide 2 in the plasma sample, ion pairs 558.0 to 698.4 *m*/*z* were monitored for quantitation, while ion pairs 558.0 to 383.2 *m*/*z*, 558.0 to 812.4 *m*/*z*, and 558.0 to 984.5 *m*/*z* were monitored for confirmation. For the SRM scan of stable isotope-labeled Peptide 2, ion pairs 561.5 to 698.4 *m*/*z*, 561.5 to 383.2 *m*/*z*, 561.5 to 812.4 *m*/*z*, and 561.5 to 984.5 *m*/*z* were monitored. Chromatograms and mass spectral data were acquired and processed using Analyst 1.6.2 software (Sciex).

### Miles assay

This assay was performed as described previously with some modifications (69). Briefly, 100 μl of 1% Evans blue dye (Nacalai Tesque) dissolved in saline was intravenously injected into mice with or without 0.2 μg/μl Gö6976, aPKC inhibitor (Fujifilm Wako Pure Chemical Corporation). At 10 min after the injection, 1 mmol/l Peptide 2 or control peptide was subcutaneously injected, and the mice were euthanized 20 min later. The subcutaneous tissue at the peptide-injected area was observed using a stereo microscope (BioTools).

### Transmigration assay

The upper inserts (5.0 μm pore size) of Chemotaxicells (Kurabo Industries, Ltd) were coated with 100 μg fibronectin (Sigma-Aldrich). HUVECs were seeded on the insert and cultured for 48 h to form an endothelial monolayer. Subsequently, U937 cells (100,000 cells/100 μl culture media) were added on the monolayer and cultured for 6 h with Peptide 2, control peptide, or histamine (Nacalai Tesque) added in the lower wells. The number of U937 cells that migrated into the lower wells was counted using the Counting chamber (ERMA Inc).

### Western blotting and pull-down assay for RhoA

These experiments were performed as described previously with some modifications (70). Briefly, total cell lysates were obtained using the RIPA lysis buffer (50 mmol/l Tris-HCl [pH 7.5], 150 mmol/l NaCl, 0.5% deoxycholate sodium, 0.1% SDS, 1% Nonidet P-40, and 100 mM PMSF). The samples were separated by SDS-PAGE and blotted on a polyvinylidene difluoride membrane. The membrane was sequentially incubated with primary Ab and horseradish peroxidase (HRP)-labeled secondary Ab, followed by treatment with HRP substrate (Luminata Forte, Millipore Corp). The target protein band was observed using the luminescent image analyzer LAS-4000 (Fujifilm Life Science). The pull-down assay was performed as described previously with some modifications (71). Briefly, the cell lysates were incubated with glutathione *S*-transferase-fused mDia Rho-binding domain and glutathione-sepharose beads (GE Healthcare) at 2 °C to collect active GTP-bound RhoA.

### Antibodies

#### Primary Abs and dilutions

Anti-CD68 rabbit polyclonal (Cat. No. ab125212) – 1:200 (Abcam), anti-nephrin guinea pig polyclonal (Cat. No. GP-N2) – 1:300 (Progen), anti-desmin rabbit monoclonal (Clone D93F5, Cat. No. 5332) – 1:300 (Cell Signaling Technology, Inc), anti-CD31 rat monoclonal (Clone SZ31, Cat. No. DIA-310) – 1:200 (Dianova), anti-podocin rabbit polyclonal (Cat. No. P0372) – 1:500 (Sigma-Aldrich), anti-RhoA rabbit monoclonal (Clone 67B9, Cat. No. 2117) – 1:1000 (Cell Signaling Technology), anti-phospho-PKC rabbit polyclonal (Cat. No. 9371) – 1:1000 (Cell Signaling Technology), and anti-glyceraldehyde-3-phosphate dehydrogenase (GAPDH) mouse monoclonal (clone 3H12, Cat. No. M171-3) – 1:1000 (Medical & Biological Laboratories).

#### Secondary Abs and dilutions

Alexa Fluor 488 or 555 goat anti-rabbit – 1:1000 (Thermo Fisher Scientific), Alexa Fluor 488 or 555 goat anti-mouse – 1:1000 (Thermo Fisher Scientific), and HRP-linked goat anti-rabbit IgG (Cat. No. NA934) or anti-mouse IgG (Cat. No. NA931) Abs – 1:2000 (GE Healthcare).

### Semiquantitative PCR for C3a receptor expression

The expression level of C3a receptor 1 (C3aR) was evaluated by semiquantitative PCR. mRNA was extracted using TRIzol RNA isolation reagent (Thermo Fisher Scientific), and cDNA was synthesized using ReverTra Ace (Toyobo). PCR was performed using rTaq DNA polymerase (TaKaRa Bio Inc). The band densities of PCR products were quantified using the ImageJ software. The mRNA expression level of C3aR was adjusted to that of *β-actin* as an internal control. Primer sequences for the experiments were as follows: *C3aR* forward 5′-AGGCAATGGGCTGGTGCTGT-3′ and reverse 5′-CAGGAAGACACTGGCAAACAT-3′; *β-actin* forward 5′-TCCTCCCCTGGAGAAGAGCTA-3′ and reverse 5′-GAATCCTGTGGCATCCAC-3′. To silence C3aR expression in HUVECs, siRNA against *C3aR* (#1: 5′-AAGCGCTTCTAGCAATTCCT-3′, #2: 5′-AAGCAGTCCATTCAGGGAATTCT-3′) was transfected into the cells with RNAiMAX (Thermo Fisher Scientific), and the cells were cultured for 2 days.

### Cell surface attachment of Peptide 2 through C3aR

FITC-conjugated Peptide 2 was generated by Biologica Co. HUVECs, in which C3aR was or was not knocked down, were incubated with the indicated concentrations of FITC-Peptide 2 for 2 h at 4 °C. Subsequently, the cells were washed with PBS three times and labeled with tetramethylrhodamine-WGA (Thermo Fisher Scientific). Following fixation with 4% paraformaldehyde, the attached peptides on the cell surface were observed using a Leica SP8 microscope (Leica Microsystems).

### Statistics

Data are expressed as mean ± standard deviation. Experiments were performed at least three times independently. Statistical significance was analyzed using Prism software (GraphPad Software) and determined by Kolmogorov–Smirnov test, one-way analysis of variance (ANOVA), or two-way ANOVA as appropriate. When ANOVA was significant, individual difference was evaluated using the Bonferroni posttest. Sample sizes and statistical significance are indicated in the figures. A value of *p* < 0.05 was considered to be statistically significant.

## Data availability

All of the data are contained within the article. The raw mass spectrometry and SRM data were deposited to the ProteomeXchange Consortium *via* the PRIDE partner repository (72), with the dataset identifiers PXD025440 for mass spectrometry and PXD025469 for SRM.

## Supporting information

This article contains supporting information.

## Conflict of interest

The authors declare that they have no conflicts of interest with the contents of this article.
