# Supplementary material for: Cardio- and reno-protective effects of dipeptidyl peptidase III in diabetic mice
Source: J Biol Chem. 2021 May 8;296:100761. doi: 10.1016/j.jbc.2021.100761 (PMC8167299; doi:10.1016/j.jbc.2021.100761)
Supplement: Supplemental Figures S1–S9 and Table S1 [file mmc6.pdf]

**Supporting Information**

# Cardio- and reno-protective effects of dipeptidyl peptidase III in diabetic mice

Masahiro Komeno, Xiaoling Pang, Akio Shimizu, Md Rasel Molla, Mako Yasuda-Yamahara, Shinji Kume, Nor Idayu A. Rahman, Joanne Ern Chi Soh, Le Kim Chi Nguyen, Mohammad Khusni B. Ahmat Amin, Nao Kokami, Akira Sato, Yoshihiro Asano, Hiroshi Maegawa, Hisakazu Ogita

This file includes:

- Supplemental Figures S1 to S9
- Supplemental Table S1
- Legends for Supplemental Movies S1 to S5

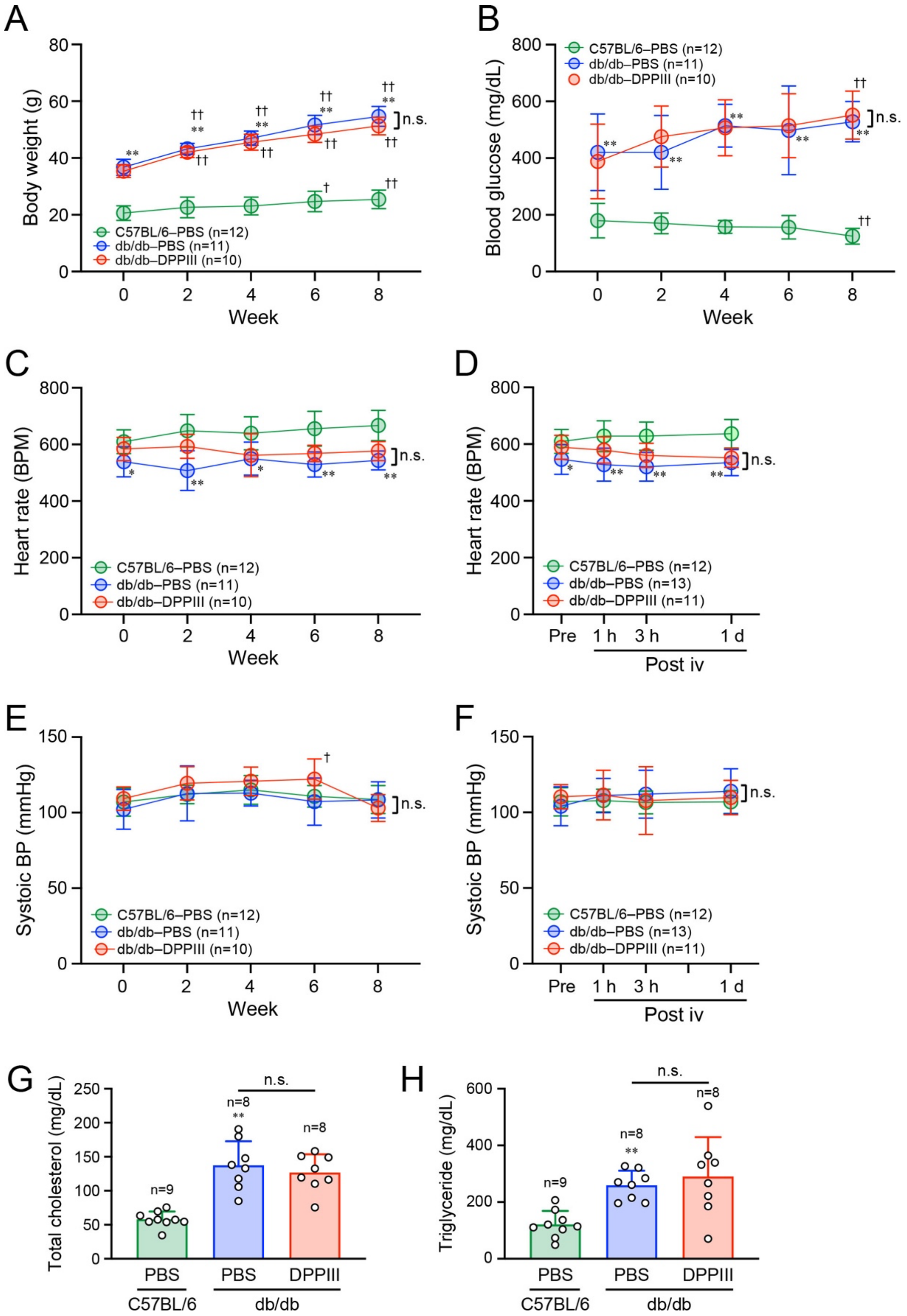


**Supplemental Figure S1. Basic measurements of experimental mice.** (**A**–**C** and **E**) Body weight (**A**), blood glucose (**B**), heart rate (**C**), and systolic blood pressure (BP) (**E**) in each group were measured every 2 weeks during the 8-week experimental period. BPM: beats per min. (**D** and **F**) Changes in heart rate (**D**) and systolic BP (**F**) pre- and post-intravenous injection (iv) of reagents were analyzed at the indicated time points. (**G** and **H**) Plasma concentration of total cholesterol (**G**) and triglyceride (**H**) after 8 weeks of treatment. * $P<0.05$ and ** $P<0.01$ vs. C57BL/6 mice; † $P<0.05$ and †† $P<0.01$ vs. 0 week. n.s.: no significance.

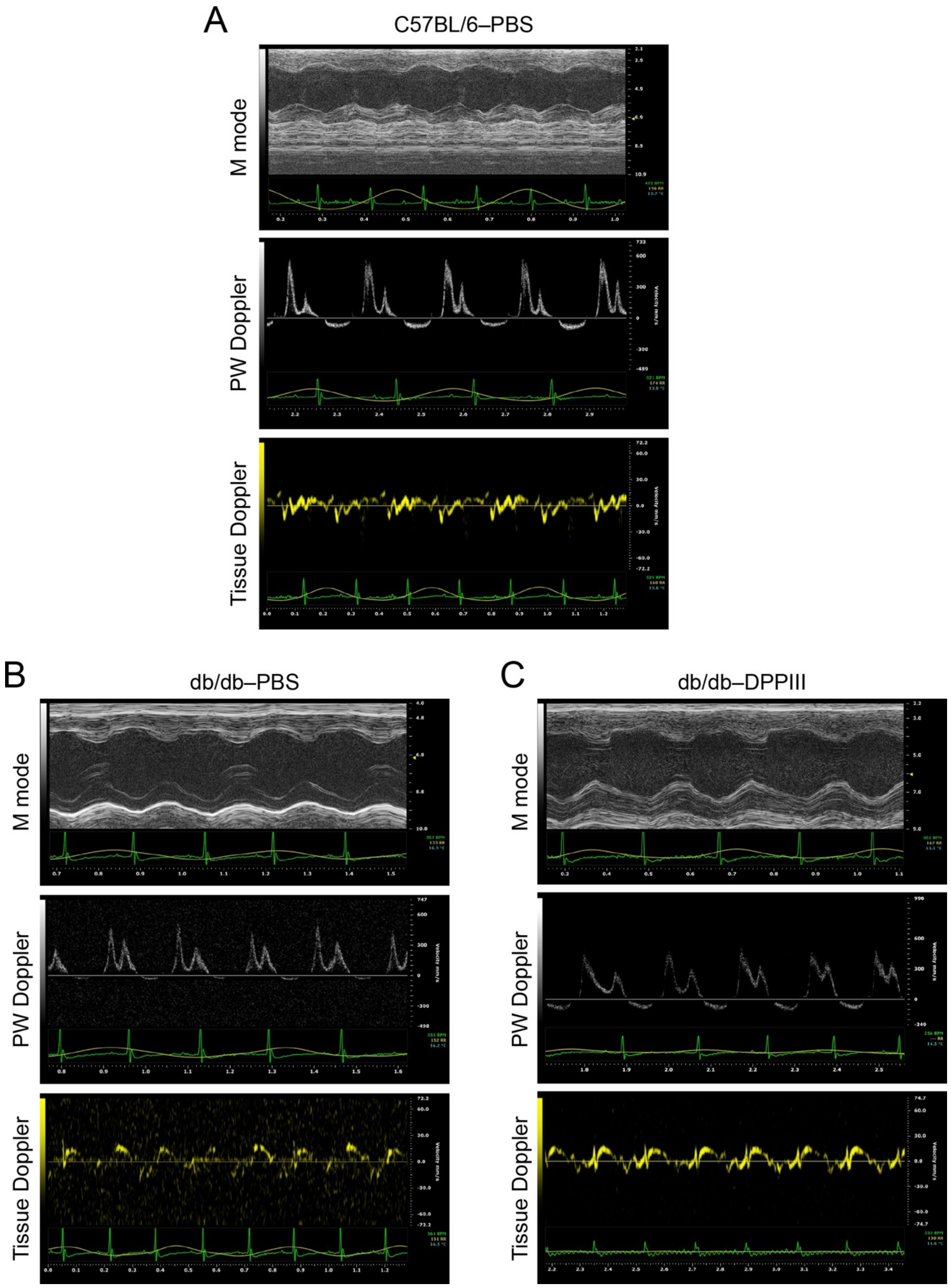


**Supplemental Figure S2. Representative images of echocardiography at 8 weeks after treatment.** (**A**) C57BL/6 mice intravenously injected with PBS (C57BL/6–PBS) (**B**) db/db mice injected with PBS (db/db–PBS) (**C**) db/db mice injected with DPPIII (db/db–DPPIII). PW: Pulsed wave.

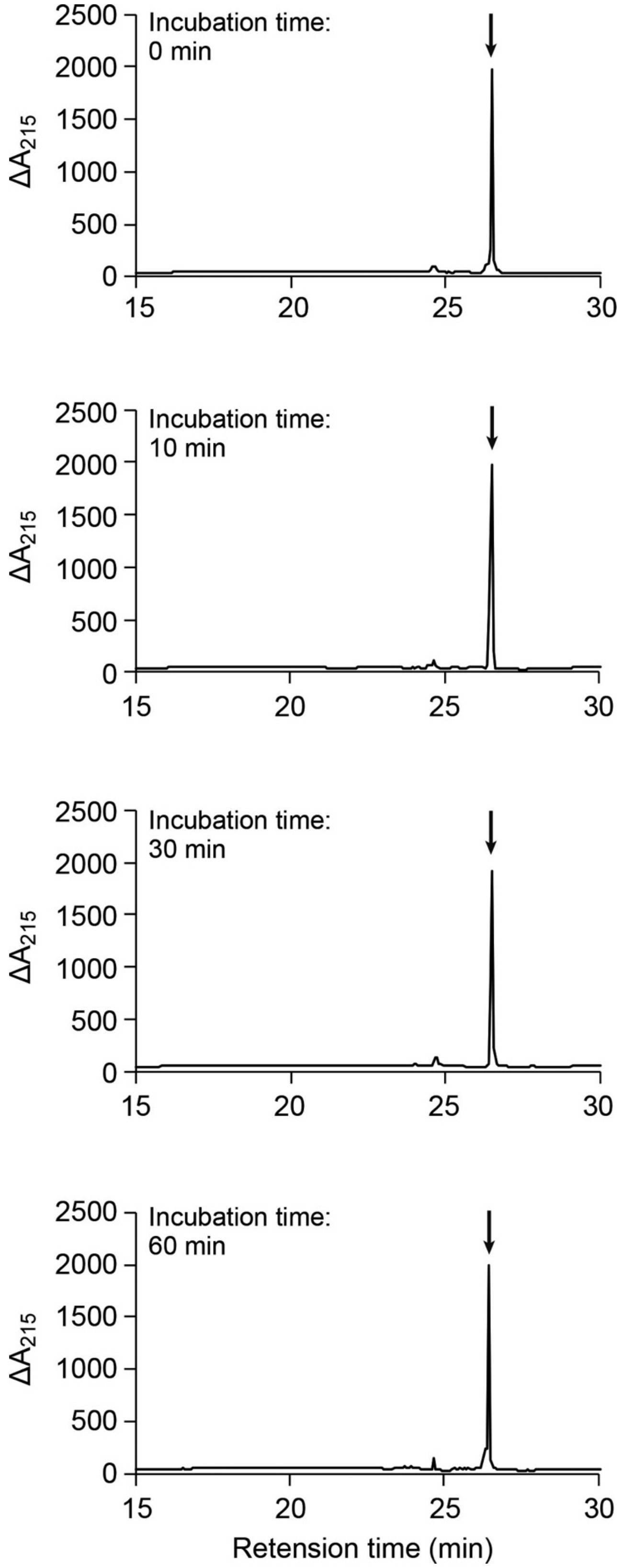


**Supplemental Figure S3. DPPIII did not cleave control peptide.** Control peptide (IAFSQYLQK) was incubated with DPPIII for the indicated time. The cleaved products were not detected by reversed-phase liquid chromatography. Arrows: control peptide.

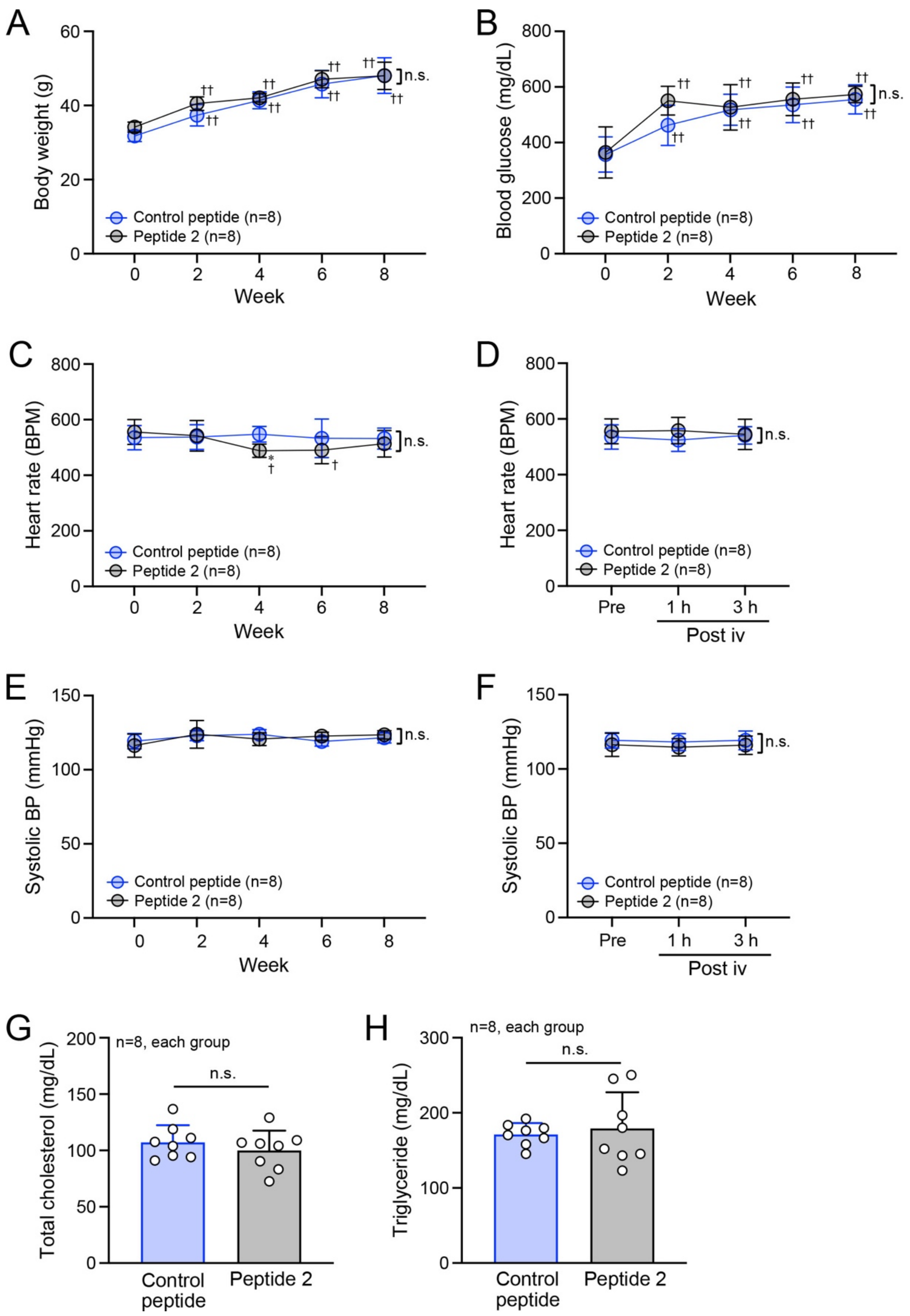


**Supplemental Figure S4. Basic measurements of diabetic mice injected with peptides.** (**A**–**C** and **E**) Body weight (**A**), blood glucose (**B**), heart rate (**C**), and systolic BP (**E**) in each group were measured every 2 weeks during the 8-week experimental period. (**D** and **F**) Changes of heart rate (**D**) and systolic BP (**F**) pre- and post-intravenous injection (iv) of reagents were analyzed at the indicated time points. (**G** and **H**) Plasma concentration of total cholesterol (**G**) and triglyceride (**H**) after 8 weeks of treatment. * $P$<0.05 vs. Control peptide; † $P$<0.05 and †† $P$<0.01 vs. 0 week. n.s.: no significance.

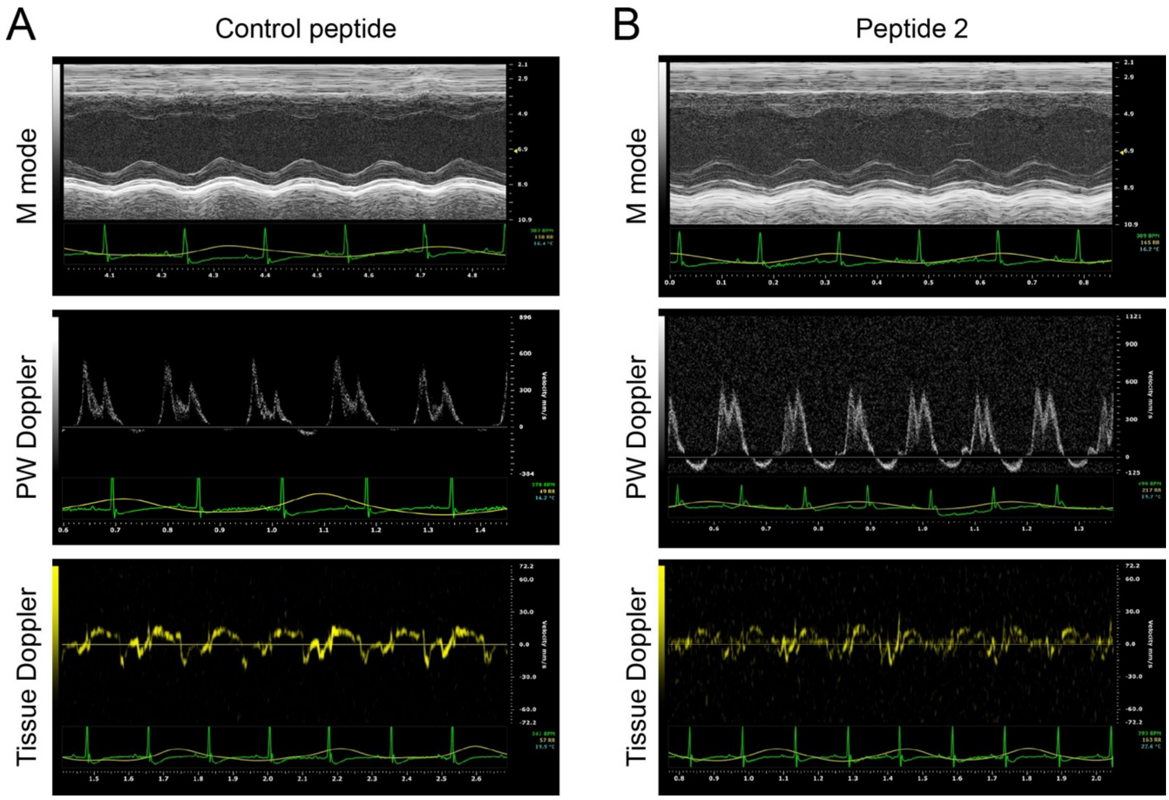


**Supplemental Figure S5. Representative images of echocardiography at 8 weeks after treatment.** (**A** and **B**) db/db mice injected with control peptide (**A**) or Peptide 2 (**B**). PW: Pulsed wave.

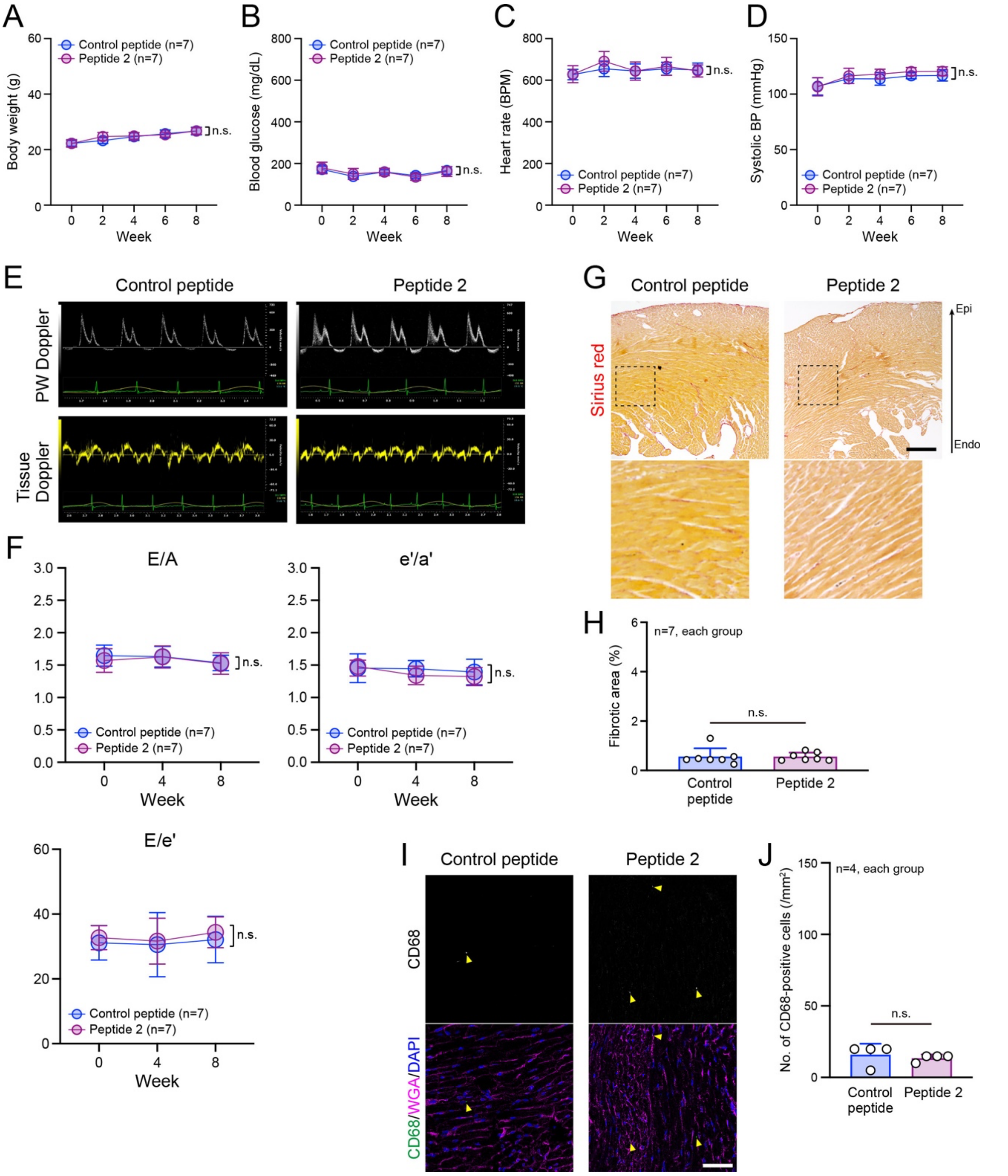


**Supplemental Figure S6. Cardiac functions after control peptide or Peptide 2 administration for 8 weeks in non-diabetic db/m mice.** (**A**–**D**) Body weight (**A**), blood glucose (**B**), heart rate (**C**), and systolic BP (**D**) were measured every 2 weeks during the 8-week experimental period. (**E**) Representative images of echocardiography after 8-week injection of control peptide or Peptide 2. PW: Pulsed wave. (**F**) Diastolic function in the heart measured by echocardiography. E/A: ratio of peak transmitral velocity of early inflow to atrial inflow, e'/a': ratio of early to atrial diastolic mitral annular velocities, E/e', ratio of peak transmitral velocity of early inflow to early diastolic mitral annular velocity. (**G**) Cardiac fibrosis detected by Sirius red staining. Dotted squares are enlarged and indicated below. (**H**) Summary graph of the percentage of fibrotic areas in the cardiac sections. (**I**) Confocal images of CD68 immunostaining in the heart. Cell membrane and nuclei were counterstained with WGA and DAPI, respectively. (**J**) Summary graph of the number of CD68-positive cells. Scale bars: 200 μm (**G**) and 50 μm (**I**). n.s.: no significance.

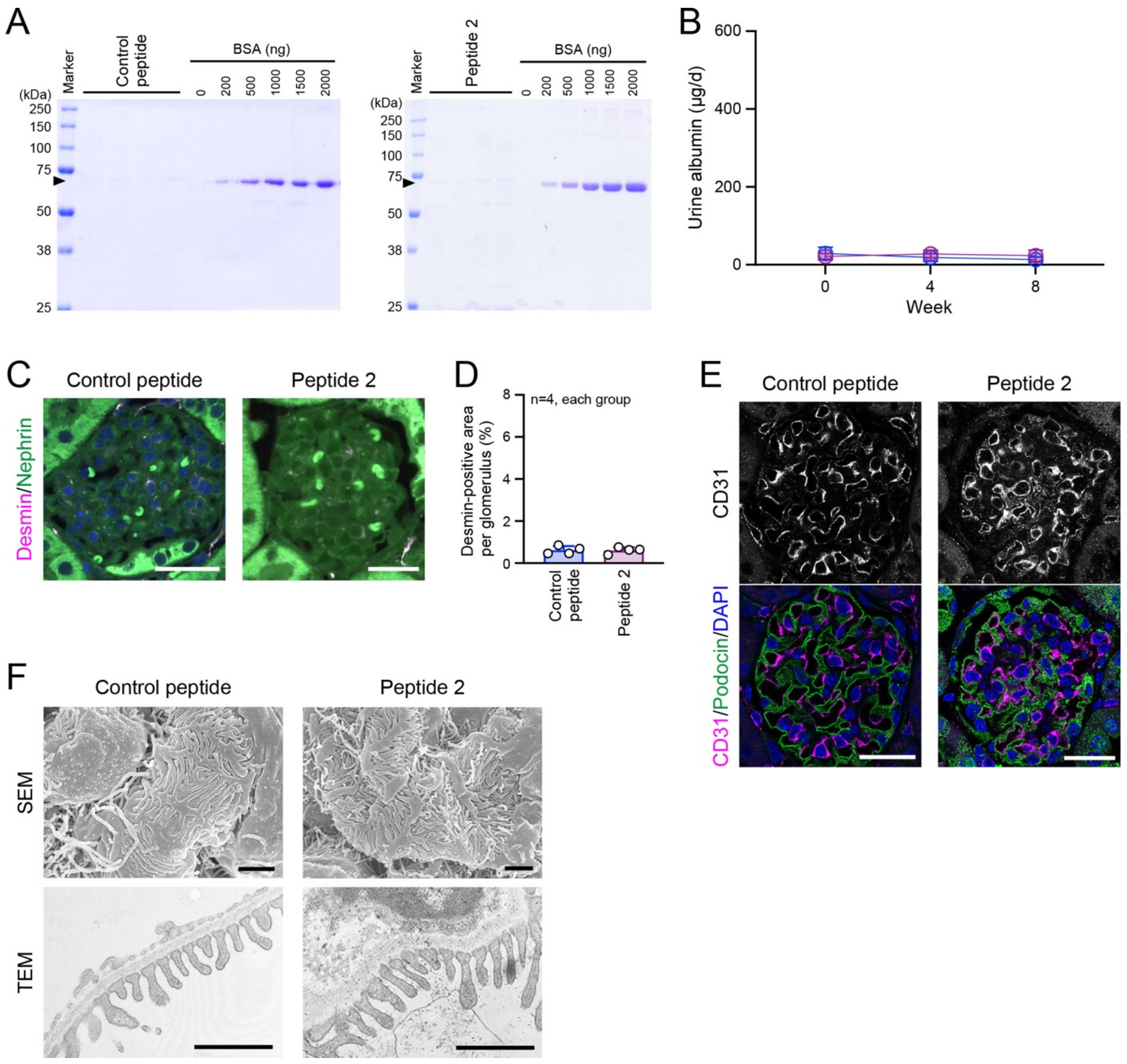


**Supplemental Figure S7. Renal functions after control peptide or Peptide 2 administration for 8 weeks in non-diabetic db/m mice.** (**A**) Representative Coomassie Brilliant Blue staining of urine samples after the 8-week administration. The urine samples from mice were collected for 24 h (1 d), and the proteins in the samples were separated by SDS-PAGE. An arrowhead indicates urine albumin. BSA was used as a control. (**B**) Graph of urine albumin excretion for 1 d at the indicated time points. (**C**) Confocal images of desmin and nephrin co-immunostaining in the glomeruli. (**D**) Summary graph of the percentage of desmin-positive areas per glomerulus. (**E**) Confocal images of CD31 and podocin co-immunostaining in the glomeruli. (**F**) Ultrastructures of the glomeruli observed by scanning electron microscopy (SEM) and transmission electron microscopy (TEM). Scale bars: 20 μm (**C** and **E**), and 1 μm (**F**). n.s.: no significance.

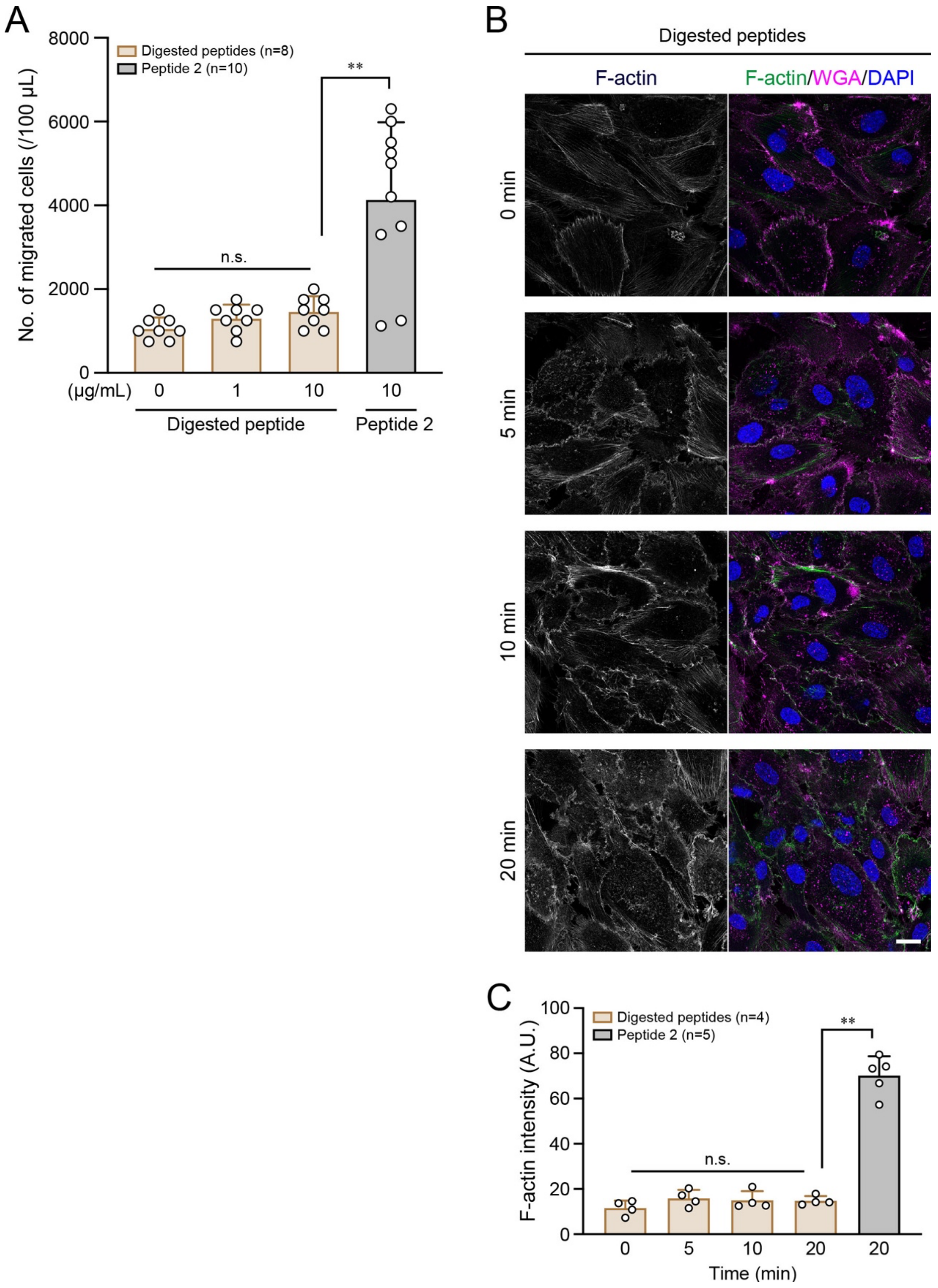


**Supplemental Figure S8. Neither chemoattractant activity nor F-actin bundling function of peptides derived from DPPIII-digested Peptide 2.** (**A**) Migration activity of U937 cells seeded on a monolayer of HUVECs in a Transwell chamber. The data for Peptide 2 stimulation are same as shown in Figure 6C. (**B**) F-actin assembly in the HUVECs treated with digested peptides for the indicated times. F-actin was stained with Alexa488-conjugated phalloidin. Cell membrane was stained with WGA. Scale bar: 20 μm. (**C**) Summary graph of phalloidin intensity examined in (**B**). The data for Peptide 2 stimulation are same as shown in Figure 6E. A.U.: arbitrary units. ** *P*<0.01. n.s.: no significance.

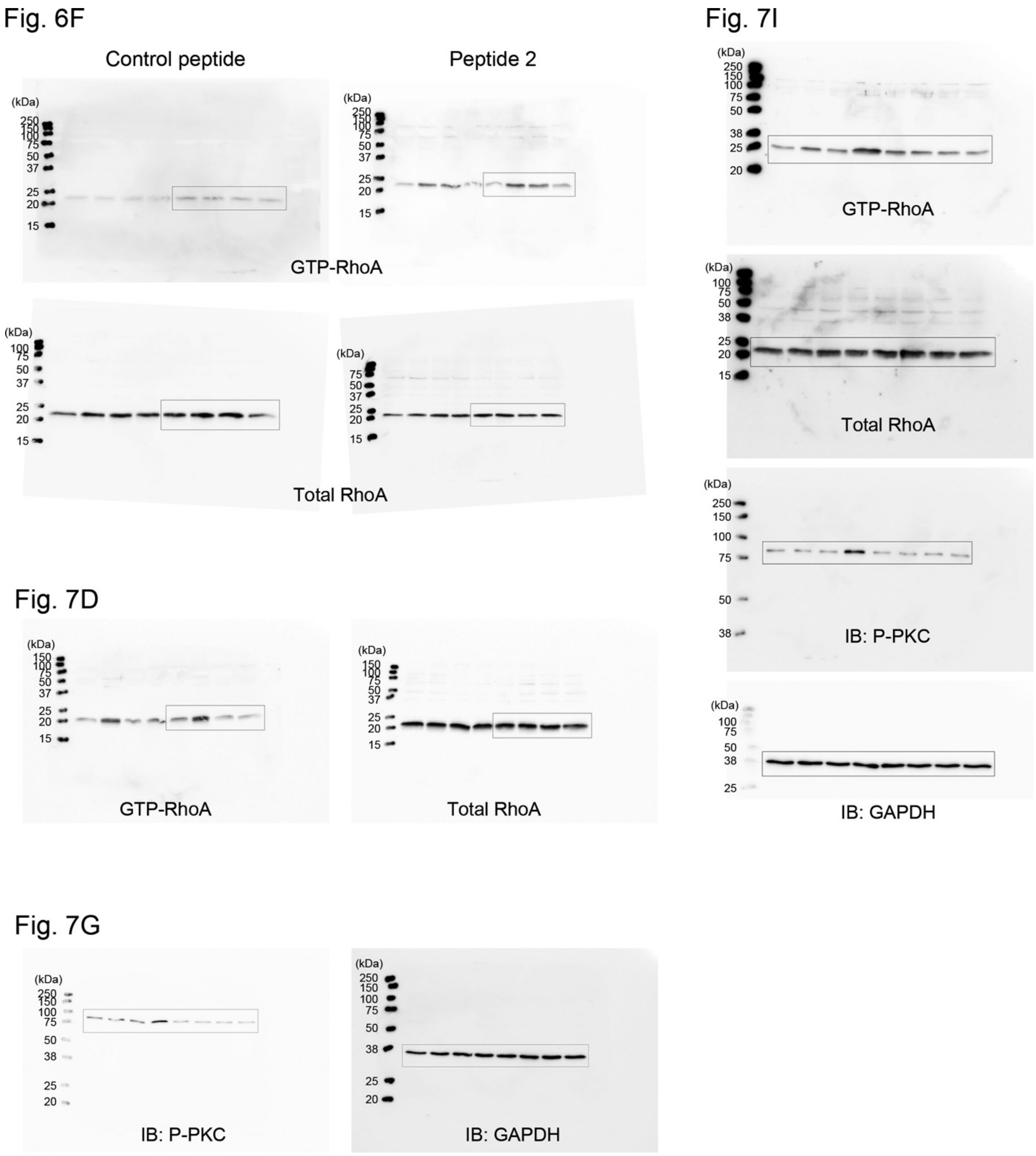


**Supplemental Figure S9. The entire images of all the western blots that were displayed in the manuscript.** The rectangles indicate the portions presented in each figure.

**Supplemental Table S1. Heart rate during echocardiography.**

| Week | 0 | 4 | 8 |
|---|---|---|---|
| C57BL/6–PBS | 437 ± 31 | 470 ± 28 | 469 ± 34 |
| db/db–PBS | 397 ± 25 | 398 ± 33 | 398 ± 35 |
| db/db–DPPIII | 387 ± 21 | 385 ± 47 | 391 ± 18 |

db/db mice

| Week | 0 | 4 | 8 |
|---|---|---|---|
| Control peptide | 370 ± 16 | 390 ± 20 | 360 ± 45 |
| Peptide2 | 393 ± 24 | 380 ± 50 | 379 ± 50 |

db/m mice

| Week | 0 | 4 | 8 |
|---|---|---|---|
| Control peptide | 410 ± 30 | 423 ± 32 | 407 ± 30 |
| Peptide2 | 417 ± 32 | 402 ± 41 | 414 ± 42 |

n=7 in each group. Data are expressed in average ± SD.

**Supplemental Movie Legends**

**Supplemental Movie S1.** Echocardiography of C57BL/6 mice after 8-week injection of PBS.

**Supplemental Movie S2.** Echocardiography of db/db mice after 8-week injection of PBS.

**Supplemental Movie S3.** Echocardiography of db/db mice after 8-week injection of DPPIII.

**Supplemental Movie S4.** Echocardiography of db/db mice after 8-week injection of control peptide.

**Supplemental Movie S5.** Echocardiography of db/db mice after 8-week injection of Peptide 2.
